# Supplementary material for: Synthesis, structures and magnetic properties of [(η9-C9H9)Ln(η8-C8H8)] super sandwich complexes
Source: Nat Commun. 2019 Jul 17;10:3135. doi: 10.1038/s41467-019-10976-6 (PMC6637141; doi:10.1038/s41467-019-10976-6)
Supplement: Supplementary file 1 — Supplementary Information [file 41467_2019_10976_MOESM1_ESM.pdf]

Supplementary Materials for

Synthesis, structures and magnetic properties of $[(\eta^9\text{-}C_9H_9)Ln(\eta^8\text{-}C_8H_8)]$ super sandwich complexes

Münzfeld *et al.*

Table of content:

Supplementary Methods 3
Supplementary Notes 12
Supplementary References 38

Supplementary Methods

General Considerations: All manipulations of air-sensitive materials were performed with the rigorous exclusion of oxygen and moisture in flame-dried Schlenk-type glassware either on a dual manifold Schlenk line, interfaced to a high vacuum (10^{-3} torr) line, or in an argon-filled MBraun glove box. Hydrocarbon solvents were predried using an MBraun solvent purification system (SPS-800) and subsequently degassed, dried and stored under vacuum over $LiAlH_4$. Tetrahydrofuran was distilled under nitrogen from potassium before storage over $LiAlH_4$. Raman spectra were obtained on a Bruker MultiRam spectrometer in flame sealed glass tubes. NMR spectra were recorded on a Bruker Avance II 300 MHz or Avance 400 MHz. ^{1}H chemical shifts were referenced to the residual proton resonance of the deuterated solvents and are reported relative to tetramethylsilane. [KC_9H_9] was prepared according to a modified procedure by Katz *et al.*[1] Complexes of the type [(η^8-C_8H_8)LnI(thf)$_n$] were prepared as reported by Mashima *et al.*[2]

Note: In order to ensure the best possible purity and reliability of all compounds, only crystalline material was isolated. Hence all yields, except that given for [K(η^9-C_9H_9)], refer to isolated crystalline samples and are generally lower compared to powder samples.

Synthesis of [K(η^9-C_9H_9)] (1): Compound **1** is prepared in a modified variant of the procedure initially reported by Katz *et al.*[1] Fine cut pieces of potassium (20.0 g; 2.00 eq; 0.512 mol) are placed in a Schlenk flask and THF (200 mL) is added. Subsequently, cyclooctatetrene (26.6 g; 28.6 mL; 1.00 eq; 0.256 mol) is slowly added under vigorous stirring at -78 °C. The reaction mixture is then allowed to warm to room temperature and stirred for 18 hours, upon which it slowly takes on a deep red color. Afterwards, the mixture is filtered through a pad of celite on a medium porosity sintered glass frit. Subsequently, the [$K_2(C_8H_8)$] containing solution is added dropwise to chloroform (250 mL) under vigorous stirring at 0 °C. Upon addition, the reaction mixture slowly turns dark brown and the precipitation of a white solid is observed. After complete addition, the reaction mixture is stirred for additional 2 hours at room

temperature and sufficient water was added to dissolve precipitated KCl. The aqueous layer is then separated and extracted with diethyl ether (100 mL). The combined organic layers are washed with water (100 mL) and brine (100 mL). Afterwards the solvents are removed under reduced pressure and the residual oily fluid is distilled (60 °C, 6.6*10-1 mbar) to obtain anti-9-chlorobicyclo-[6.1.0]-nonatriene as a turquoise liquid. Yield: 13.3 g (45 %).

In a second step small pieces of potassium (8.39 g; 5.00 eq; 0.164 mol) and THF (400 mL) are placed in Schlenk flask. Anti-9-chlorobicyclo-[6.1.0]-nonatriene (5.00 g; 1.00 eq; 0.033 mol) is added dropwise under vigorous stirring at -78 °C. Subsequently, the reaction mixture is allowed to warm to room temperature and further stirred for 4 days. A color change to black and the precipitation of an off-white solid is observed. Afterwards, the mixture is concentrated to approximately 200 mL under vacuum and filtered over a pad of celite on a medium porosity sintered glass frit to remove excess potassium. Afterwards, volatiles are removed under reduced pressure and the residual oily solid is excessively washed with toluene until the washing solution remains colorless. The residual solid is then dried under reduced pressure to obtain of **1** as a dark grey powder. Yield 3.30 g (64 %). Single crystals suitable for X-ray diffraction were obtained by recrystallisation of the amorphous product from hot DME and storage at -20 °C.

^{1}H-NMR (400 MHz, 298 K, THF-d_8): δ [ppm] = 7.05 (s, 9 H, C-H). NMR-data are consistent with literature values.[1]

Synthesis of [(η^8-C_8H_8)LnI(thf)$_n$] (2a-d): The synthesis of **2a-d** was conducted according to a procedure published by Mashima *et al.*[2] for Ln= (La, Ce, Pr, Nd, Sm). In a typical reaction, THF (40 mL) is added to 4.70 mmol (1.00 eq) finely grated lanthanide metal in a Schlenk flask sealed with a *J. Young* valve. Subsequently, 2.35 mmol (0.50 eq) iodine and 4.70 mmol (1.00 eq) cyclooctatetraene, as well as one drop of mercury are added to the stirred suspension. The reaction mixture is then sonicated at 80 °C for 24 h and afterwards stirred at 80 °C in an oil bath until most of the lanthanide metal had vanished (only small amounts of residual metal are still visible). Subsequently, unreacted metal and amalgam are removed by filtration over a sintered glass frit. The crude product is recrystallized from hot THF to yield the desired clean products as crystalline solids. In the case of Nd and Sm, the reaction is usually finished after 48 h as described by Mashima *et al.*[2] However, for Dy and Er reaction times of three to four weeks are observed depending on the amount of mercury added. X-ray quality single crystals were obtained by recrystallisation from hot THF.

[(η^8-C_8H_8)NdI(thf)$_3$] (**2a**):

Yield: 2.37 g (97 %).

Cell found by X-ray-diffraction in agreement with the reported Structure by Anwander *et. al.*:[3]

a = 12.9151, b = 21.9914, c = 15.101, α = γ = 90°, β = 90.658°.

[(η^8-C_8H_8)SmI(thf)$_2$] (**2b**):

Yield: 1.98 g (80 %).

^{1}H-NMR (400 MHz, 298 K, THF-d_8): δ [ppm] = 8.02 (s, 8 H, C_8H_8). NMR data are consistent with literature values.[2]

[(η^8-C_8H_8)DyI(thf)$_2$] (**2c**):

Yield: 1.36 g (54) %.

Raman (solid state, sealed ampule): $\tilde{\nu}$ [cm^{-1}] = 3042 (vw), 2982 (vw), 2891 (vw), 1496 (vw), 752 (s), 383 (vw), 239 (m), 99 (vw).

[(η^8-C_8H_8)ErI(thf)$_3$] (**2d**):

Yield: 1.07 g (42 %).

Raman (solid state, sealed ampule): $\tilde{\nu}$ [cm^{-1}] = 3036 (vw), 2976 (vw), 2897 (vw), 1498 (vw), 752 (s), 378 (vw), 230 (m), 106 (vw).

Cell found by X-ray-diffraction in agreement with the reported Structure by Rinehart *et al.*:[4]

a = 8.3977, b = 9.5990, c = 21.4292, α = γ = 90°, β = 96.484°.

Synthesis of [(η^9-C_9H_9)Ln(η^8-C_8H_8)] (3a-d): A mixture of 0.60 mmol (1.00 eq) of the corresponding precursor [(η^8-C_8H_8)LnI(thf)$_n$] **2a-d** and 0.60 mmol (1.00 eq) of **1** are placed in a Schlenk tube. The mixture is suspended in toluene (20 mL) and heated under reflux for 30 min with vigorous stirring. By this time, the solution adopts a characteristic color depending on the central lanthanide ion (Nd: yellow, Sm: green, Dy: orange, Er: red) and the formation of an off-white solid is observed. Subsequently, the reaction mixture is filtered through a PTFE-filter and the solvent is removed under vacuum. X-ray quality single crystals were obtained by recrystallisation from hot toluene.

Due to the high thermal stability of compounds **3a-d**, elemental analysis yielded reproducibly too low carbon values. Thus, High-Resolution EI-MS spectra were recorded.

[(η^9-C_9H_9)Nd(η^8-C_8H_8)] (**3a**):

Yield: 79.0 mg (36 %).

Raman (solid state, sealed ampule): $\tilde{\nu}$ [cm^{-1}] = 3045 (m), 3008 (m), 1518 (w), 1497 (w), 748 (s), 677 (s), 386 (w), 289 (w), 242 (m).

EI-MS: (70 eV, QT = 200 °C): *m/z* [%] = 363.040 [M]$^+$ (100).

[(η^9-C_9H_9)Sm(η^8-C_8H_8)] (**3b**):

Yield: 71.0 mg (32 %).

Raman (solid state, sealed ampule): $\tilde{\nu}$ [cm^{-1}] = 3041 (w), 3006 (w), 1518 (w), 1496 (w), 748 (s), 678 (s), 384 (w), 286 (w), 236 (s).

EI-MS: (70 eV, QT = 220 °C): *m/z* [%] = 373.049 $[M]^+$ (17.8).

[(η^9-C_9H_9)Dy(η^8-C_8H_8)] (**3c**):

Yield: 71.0 mg (31 %).

Raman (solid state, sealed ampule): $\tilde{\nu}$ [cm^{-1}] = 3044 (w), 2999 (vw), 1514 (w), 1492 (w), 751 (s), 693 (m), 392 (w), 269 (m), 248 (m).

EI-MS: (70 eV, QT = 200 °C): *m/z* [%] = 385.072 $[M]^+$ (17.4).

[(η^9-C_9H_9)Er(η^8-C_8H_8)] (**3d**):

Yield: 75.0 mg (32 %).

Raman (solid state, sealed ampule): $\tilde{\nu}$ [cm^{-1}] = 3043 (m), 3000 (vw) 1514 (w), 1492 (w), 751 (s), 692 (m), 391 (w), 269 (m), 247 (m).

EI-MS: (70 eV, QT = 180 °C): *m/z* [%] = 387.049 $[M-2H]^+$ (56.9).

Raman Spectra of 3a-d:

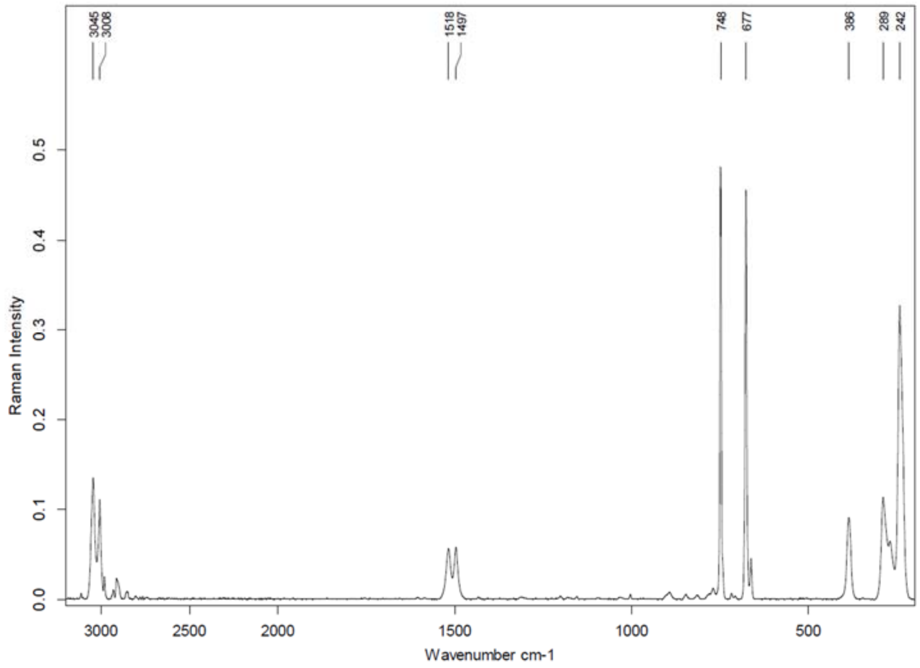

Supplementary Figure 1: Raman spectrum of **3a**.

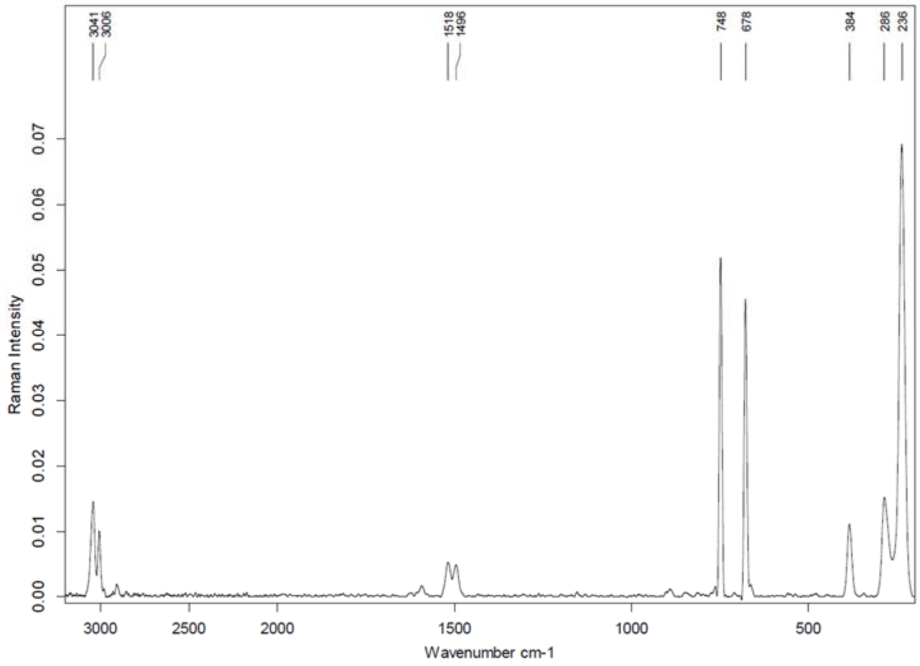

Supplementary Figure 2: Raman spectrum of **3b**.

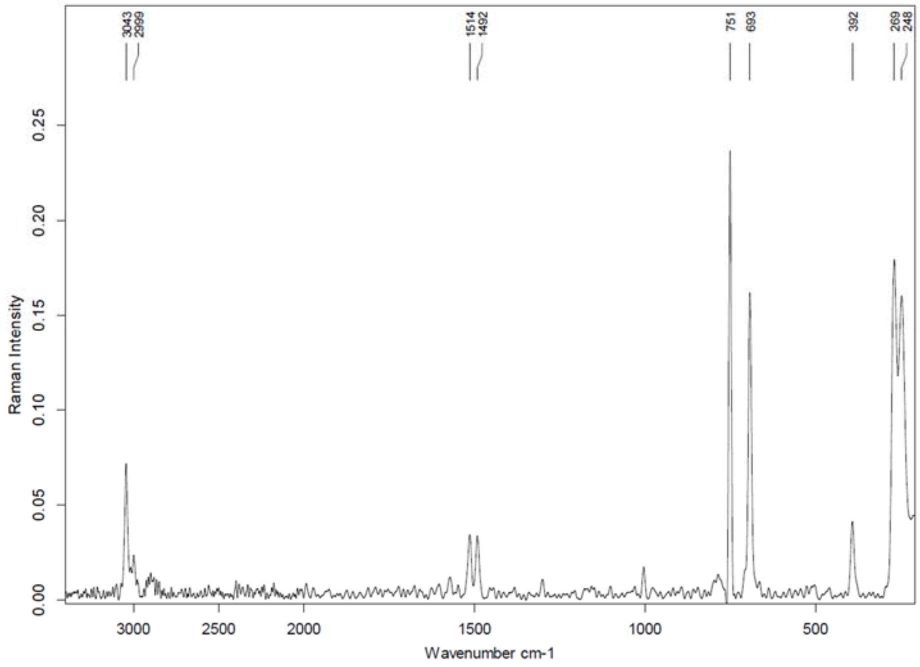

Supplementary Figure 3: Raman spectrum of **3c**.

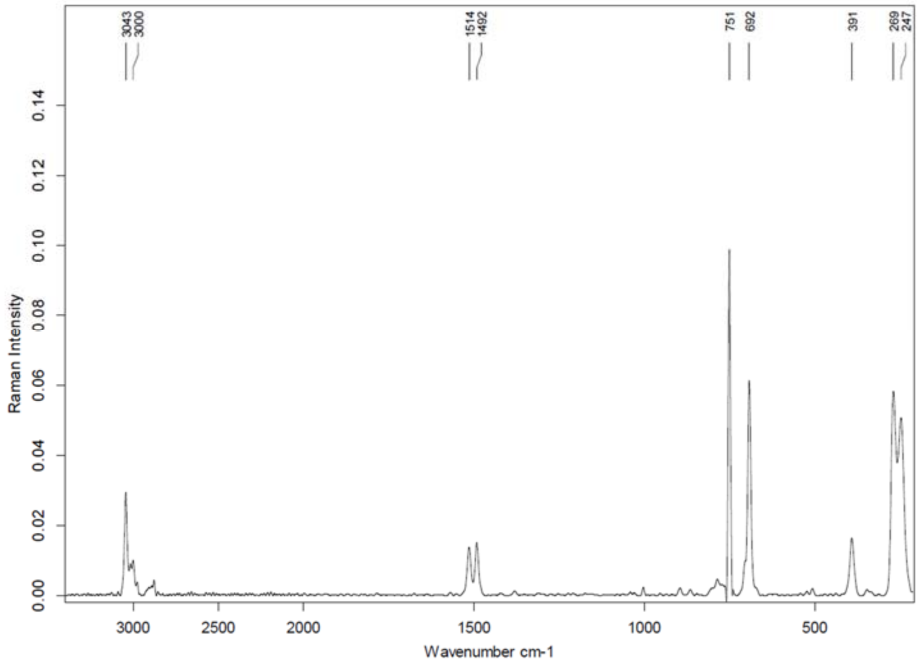

Supplementary Figure 4: Raman spectrum of **3d**.

High-Res EI-MS spectra of 3a-d:

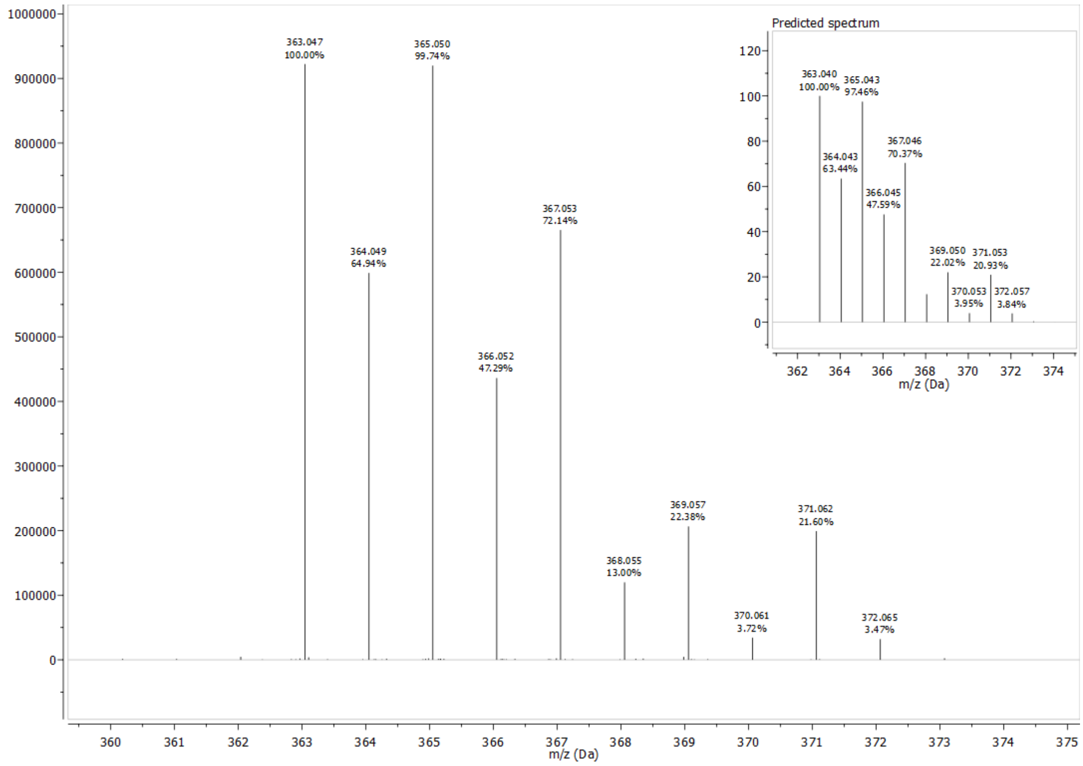

Supplementary Figure 5: Cutout of the EI-MS spectrum of **3a**. Calculated mass for $[M]^{+}$: *m/z* = 363.040; found: 363.047.

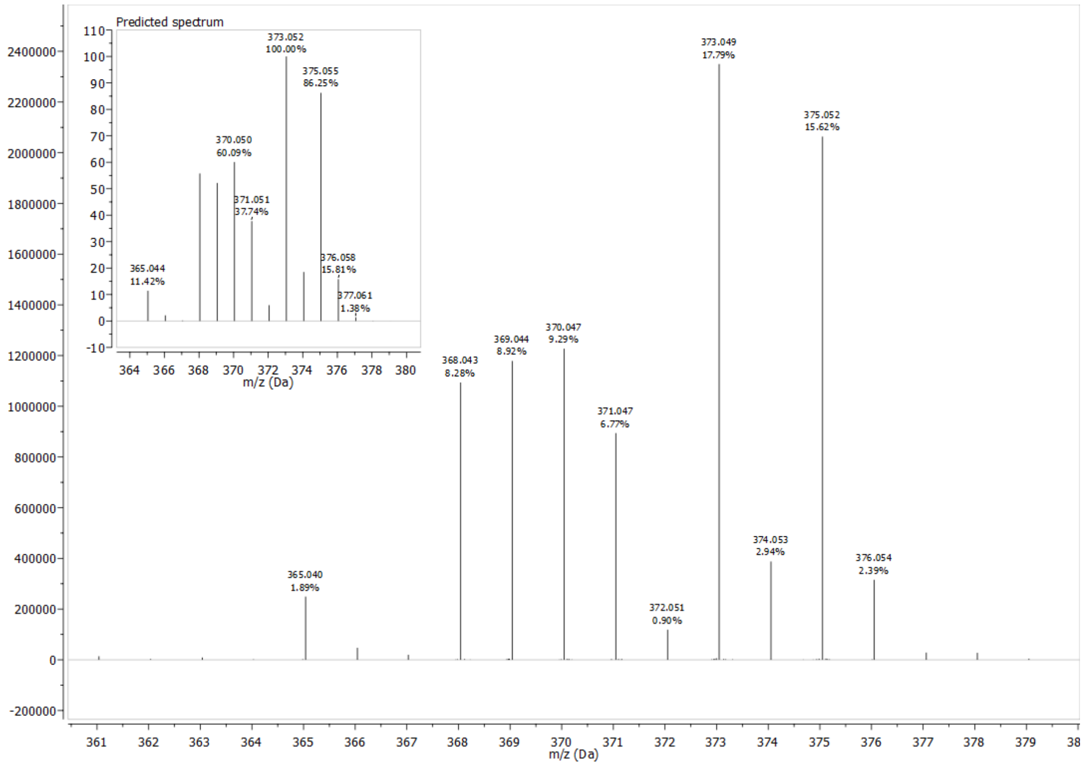

Supplementary Figure 6: Cutout of the EI-MS spectrum of **3b**. Calculated mass for $[M]^{+}$: *m/z* = 373.052; found: 373.049.

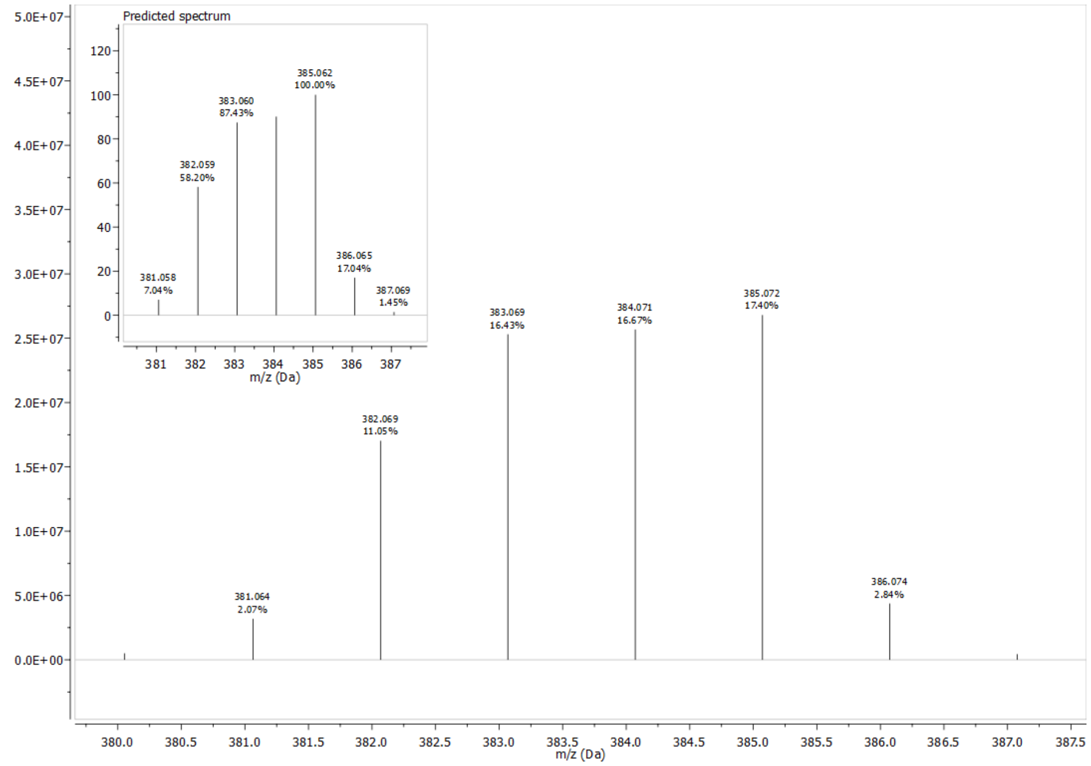

Supplementary Figure 7: Cutout of the EI-MS spectrum of **3c**. Calculated mass for $[M]^+$: *m/z* = 385.062; found: 385.072.

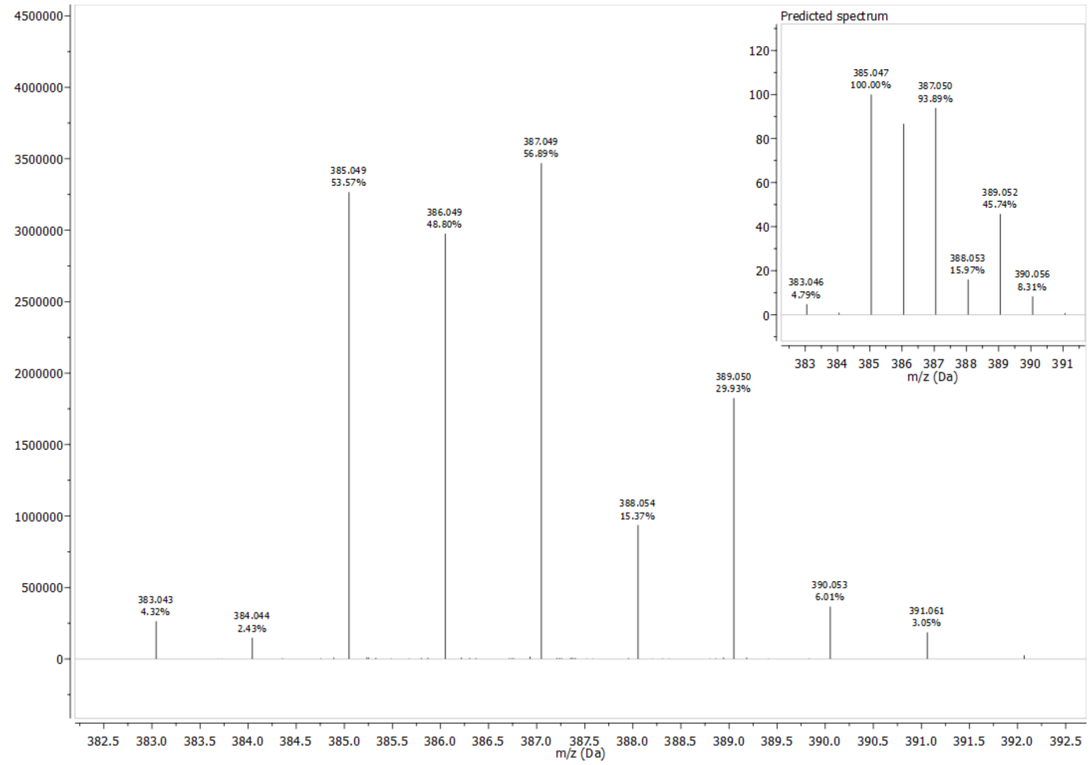

Supplementary Figure 8: Cutout of the EI-MS spectrum of **3d**. Calculated mass for $[M-2H]^+$: *m/z* = 385.047; found: 385.049.

Supplementary Notes

Computational Details: The quantum chemical RI-DFT calculations were performed by means of the program system TURBOMOLE using the RI-BP86 functional.[5-10]

The basis sets for C, H, N, Nd, Dy, Sm and Er were of def-SV(P) quality.[11,12]

For the lanthanide atoms, relativistic corrected effective core potentials of def-ecp quality have been used to simulate the 28 inner electrons. The geometry optimization by means of analytically derived gradients using redundant internal coordinates lead to molecules of C_s symmetry. Several electronic occupations have been tested to find the ground state electronic configuration of the molecules under discussion. The theoretical vibrational spectra were obtained by calculation of the second derivatives using the module AOFORCE.[13]

For the calculated molecules, in each case one very small imaginary frequency (less than 15i cm^{-1}) was found. The associated modes did not, however, involve movements of interest, and so the optimized structures and other computed vibrational details were used in the discussion of the vibrational spectra.

Details for Raman band assignment: According to the normal coordinate method of Wilson, Decius and Cross the most prominent G matrix element which is necessary to determine the vibrational frequency of the fully symmetric ring breathing mode ν_{sym} in aromatic C_nH_n-monocyclic ligand systems is G = 2 μ_C·(1 + cos Θ).[14] Assuming equally strong C-H and C-C (F) bonding force constant values in these ligand systems,[15,16] this vibrational frequency depends in a first approximation only on the inner angle Θ(C-C-C) = (n-2)/n·180° of the n-egded polygon built up by $C_nH_n^x$. Due to the correlation G·F = $5.891\cdot10^{-7}\ \nu_{sym}^2$ the term $\nu_{sym}^2/(1+\cos\Theta)$ should give a constant value of about $19.76\cdot10^5$ and leads to the following mathematical expression:[16,17]

Supplementary Equation 1: Approximation for the fully symmetric ring breathing mode´s energy of an aromatic monocyclic system (C_nH_n).[16,17]

$$\nu_{sym}(C_nH_n{}^x)[cm^{-1}] = \sqrt{19.76\cdot10^5\cdot(1+\cos\left[\left(1-\frac{2}{n}\right)\cdot180°\right]}$$

This situation of comparable bond strength is nicely confirmed for all aromatic monocycle systems for n = 5, 6, 7, 8, 9: $\nu_{sym}(C_5H_5^-)$ = 1095[18] (calc. 1168), $\nu_{sym}(C_6H_6)$ = 992[19] (calc. 994), $\nu_{sym}(C_7H_7^+)$ = 868[15] (calc. 863), $\nu_{sym}(C_8H_8^{2-})$ = 754 (this work, calc. 761) and $\nu_{sym}(C_9H_9^-)$ = 680 cm^{-1} (this work, calc. 680 cm^{-1}).

Structural data in Cartesian coordinates are summarized in a separated file.

Supplementary Table 1: Electronic ground state, electron distribution and total energy of **3a-d**.

	electronic ground state	Occupation of electrons	E_{tot} [Hartree]
**3a**	^{4}a’	1 a’ 2 a’’	-1219.1898893990
**3b**	^{6}a’	3 a’ 2 a’’	-1315.8807199140
**3c**	^{6}a’’	2 a’ 3 a’’	-1543.5606906650
**3d**	^{4}a’	1 a’ 2 a’’	-1673.5036248030

Supplementary Table 2: Vibrational frequencies and relative Raman intensities for **3a** and **3b**.

**3a**			**3b**		
Irrep	ν [cm^{-1}]	Raman Int.	Irrep	ν [cm^{-1}]	Raman Int.
**a"**	-8.69	0	a"	-6.82	0
**a'**	55.67	15.774849	a'	55.11	4.68397071
**a"**	72.73	9.60963279	a"	59.32	3.41941032
**a"**	130.81	3.38158733	a'	128.46	100
**a'**	131.07	100	a"	130.27	2.16025743
**a'**	131.75	0.86849468	a"	132.73	50.061167
**a'**	132.33	89.4602783	a'	133.17	50.2517597
**a"**	155.19	46.0818555	a'	134.66	0.86943957
**a'**	215.22	18.9039616	a'	197.72	1.64808609
**a'**	221.67	31.9163306	a"	202.69	10.2162119
**a"**	239.67	10.0495919	a'	202.92	8.69031789
**a'**	243.14	1.02836578	a"	233.55	0.21174406
**a"**	253.67	26.2721186	a'	236.04	0.19054128
**a'**	276.88	12.9234124	a"	276.53	5.8533234
**a"**	277.09	12.6274048	a'	278.11	5.81467298
**a'**	362.89	5.66215773	a'	354.25	2.78345123
**a"**	367.22	5.57455184	a"	357.51	2.71820648
**a'**	397.56	0.1802906	a"	397.44	3.35E-04
**a"**	399.4	0.06716902	a'	397.6	0.00464328
**a'**	496.44	0.0039326	a'	490.78	0.1157465
**a"**	506.81	0.08022693	a"	523.24	0.0058902
**a"**	572.78	0.11113598	a"	571	0.00319371
**a'**	593.54	0.00104795	a'	595.49	0.25698278
**a"**	596.55	0.02323331	a"	599.35	0.00700628
**a'**	629.1	0.00163611	a'	628.65	0.00115026
**a"**	631.07	0.01273527	a"	634.32	0.00151834
**a'**	649.42	0.04016556	a'	652.22	0.17606333
**a'**	678.57	22.9964687	a'	678.44	10.3424552
**a'**	693.74	3.02583602	a'	687.52	3.24568551
**a'**	748.25	0.38455126	a"	744.02	0.32031984
**a"**	749.65	0.32633194	a'	746.77	0.24199156
**a'**	751.6	0.06675916	a'	748.27	0.44974292
**a'**	752.66	21.2815757	a'	751.5	9.98572257
**a"**	761.1	0.3206472	a"	758.14	0.04851515
**a'**	765.57	0.10629643	a"	764.53	0.1010815
**a"**	804.72	0.9907763	a'	766.67	0.0412088
**a'**	817.79	0.01790923	a"	825.46	0.00704227
**a"**	833.11	0.410148	a'	826.5	0.00737195
**a'**	854.73	0.49337167	a'	854.58	0.31827208
**a"**	860.4	0.83129113	a"	854.85	0.30795348
**a'**	870.31	0.00768153	a'	871.03	0.00345319
**a"**	871.93	0.03852401	a"	874.57	0.00582531
**a'**	888.61	0.00229772	a'	888.66	0.00724252
**a"**	888.77	6.86E-04	a"	888.77	0.00388517
**a'**	890.04	0.00471524	a'	889.18	0.00492359
**a"**	895.56	0.01473998	a'	902.77	0.5761307
**a'**	903.45	0.58412288	a"	902.96	0.56038686

a"	912.82	0.15202709	a"	914.78	0.00130936
a"	924.22	0.88653687	a"	929.54	1.15E-04
a'	930.54	0.00310669	a'	932.54	0.00274586
a"	936.95	0.48762133	a"	953.85	6.32E-04
a'	961.21	5.01E-04	a'	961.43	0.02163546
a"	961.6	0.00482353	a"	963.13	0.0030486
a"	974.06	0.56966984	a"	986.31	0.07959594
a"	1174.41	0.03517975	a'	1174.78	0.01393499
a'	1174.9	0.03679254	a"	1175.08	0.03256564
a'	1179.96	0.44053799	a'	1177.87	0.16788557
a"	1180.45	0.47231924	a"	1181.02	0.19999911
a'	1297.51	0.00107996	a'	1300.04	0.00136239
a'	1305.92	0.00414326	a'	1306.3	0.00509911
a"	1307.33	0.00930803	a"	1309.96	0.0027487
a"	1367.28	1.70E-04	a"	1370.09	8.12E-05
a"	1370.22	3.75E-04	a'	1370.68	0.00151898
a'	1371.1	0.02902958	a"	1371.18	0.00230466
a"	1399.28	7.70E-04	a"	1398.72	4.46E-05
a'	1400.6	2.81E-04	a'	1402.45	8.57E-04
a"	1402.69	3.93E-05	a"	1403.85	2.70E-04
a'	1421.36	0.30537406	a'	1423.43	0.31969044
a"	1422.5	0.26236999	a"	1424.74	0.2942574
a"	1441.48	0.12135731	a"	1441.88	0.09946191
a'	1442.57	0.14023503	a'	1443.73	0.10080847
a'	1504.35	1.55097158	a'	1449.12	0.40855983
a"	1505.21	1.32910098	a'	1504.09	0.87785224
a"	1511.49	3.29890203	a"	1508.71	0.68307537
a'	1512.61	3.13793103	a"	1511.72	1.38208961
a'	1515.49	0.09676784	a'	1512.31	0.0272927
a"	1519.24	0.01622658	a'	1513.2	1.35793309
a'	1523.83	0.03586053	a"	1517.75	0.10640923
a"	1536.18	0.11700982	a'	1526.57	1.01941386
a'	1549.86	0.73986454	a"	1579.14	0.00969044
a'	1578.25	0.14496266	a'	1579.36	0.00829025
a"	1580.01	9.61E-04	a"	1692.9	0.01522605
a'	3047.58	0.00211919	a"	3036.14	0.23692889
a"	3047.61	4.60E-05	a'	3037.31	0.27421148
a"	3051.8	0.01404068	a'	3049.46	0.03873287
a'	3054.59	0.00243261	a"	3052.58	0.00296243
a"	3054.84	0.00213641	a"	3054.42	0.00167391
a'	3055.33	0.00353133	a'	3056.4	0.00738604
a"	3056.34	0.01085387	a"	3059.84	0.01666108
a'	3066.95	0.03935395	a"	3061.18	1.42295623
a"	3067.15	0.65169905	a'	3065.78	1.4319717
a'	3067.72	7.69831928	a"	3068.4	1.67357233
a"	3068.13	7.03857361	a'	3068.47	2.0217453
a'	3080.33	0.91308878	a"	3076.94	0.65420279
a"	3080.88	0.16711499	a'	3077.99	1.01291598
a'	3081.34	0.11776624	a"	3081.64	0.23592717
a"	3083.42	0.51041044	a'	3082.21	0.28998458

a'	3086.74	7.9510642	a'	3084.32	4.03680655
a'	3088.43	13.673504	a'	3089.35	5.31999184

Supplementary Table 3: Vibrational frequencies and relative Raman intensities for **3c** and **3d**.

	**3c**			**3d**	
**Irrep**	*v* [cm^{-1}]	Raman Int.	Irrep	*v* [cm^{-1}]	Raman Int.
a"	-14.96	0	a"	-3.63	0
a"	14.49	91.0083924	a"	67.15	0.21597388
a'	31.05	100	a'	69.08	0.25527621
a'	57.06	6.78278345	a"	94.13	88.9568449
a"	63	5.48558775	a'	98.08	80.9545717
a'	77.91	28.9389456	a'	105.73	2.02754925
a"	81.8	5.5417853	a"	114.54	1.99130403
a'	123.7	28.5070433	a'	130.94	100
a'	195.31	3.02028322	a'	190.35	0.89271668
a"	217.51	0.63745139	a"	193.49	14.2835751
a'	218.61	2.97422728	a'	196.34	12.9343473
a'	222.37	2.54342284	a'	227.59	0.31929711
a"	227.03	1.86309756	a"	239.01	0.27934437
a'	255.39	1.55349002	a'	263.94	6.82903947
a"	256.51	1.24805076	a"	264.68	6.80351086
a'	338.36	0.73177767	a'	348.82	3.34406798
a"	341.2	0.65972571	a"	354.71	3.27027856
a"	376.55	0.0033161	a"	391.54	0.00854018
a'	381.83	0.16067474	a'	391.75	0.01234557
a'	483.65	0.01055453	a'	494.84	0.31048207
a"	500.54	0.0034346	a"	501.57	0.02080149
a"	562.57	0.00384067	a"	556.53	0.01413473
a"	585.66	0.00202106	a'	593.54	0.37344844
a'	591.46	0.01701632	a"	595.73	0.00313939
a"	621.03	0.00125313	a"	624.06	1.70E-04
a'	621.74	0.02916187	a'	625.49	0.00221431
a'	647.44	0.01732688	a'	649.13	0.08819369
a'	679.29	2.61734681	a'	679.69	11.5771
a'	693.73	0.5072238	a'	690.35	2.66726294
a'	744.13	0.03133702	a"	739.12	0.24704121
a"	744.87	0.02398165	a'	749.62	0.69276359
a'	751.87	0.66224269	a"	750.15	0.01456583
a"	753.16	0.01967807	a'	750.28	2.17216867
a'	753.93	1.96177543	a'	753.72	6.91039817
a"	759.9	0.00577737	a"	760.81	0.02130512
a'	766.22	0.0116636	a'	766.46	0.16934221
a'	818.2	0.00829543	a'	808.79	0.03441311
a"	819.35	5.18E-04	a"	831.51	0.00117912
a'	854.63	0.07052606	a"	855.71	0.32529588
a"	858.15	0.06310687	a'	856.32	0.32458324
a'	866.49	0.01405925	a'	866.64	0.01378482
a"	867.36	9.07E-04	a"	871.5	0.00435601
a"	885.4	2.51E-04	a'	887.45	0.71802158
a'	885.8	0.00249837	a"	888.03	0.02589395

a'	887.56	0.00337989	a'	891.42	0.07048874
a"	893.03	5.09E-04	a"	891.59	0.00550723
a"	900.23	0.11772828	a"	901.79	0.47252291
a'	906.2	0.10013956	a'	902.45	0.54235224
a"	911.71	4.45E-04	a"	915.71	0.0174972
a'	923.79	2.33E-04	a'	926.16	0.04043895
a"	927.75	1.84E-05	a"	929.19	9.55E-04
a"	954.42	3.42E-04	a"	956.82	0.0108281
a"	956.13	3.73E-04	a"	958.75	0.03267753
a'	960.04	4.34E-04	a'	959.1	0.06071931
a'	1172.76	0.01777238	a"	1167.62	0.05037707
a"	1172.89	0.00281771	a"	1174.7	0.04954716
a"	1177.54	0.02811329	a'	1178.94	0.02977286
a'	1178.04	0.10875528	a'	1190.24	0.28635347
a'	1297.34	1.05E-04	a'	1299.14	0.01342931
a"	1306.89	1.40E-04	a'	1307.67	0.00365871
a'	1307.05	4.94E-05	a"	1308.03	0.00534152
a"	1366	9.10E-06	a"	1368.13	1.56E-04
a"	1370.2	1.79E-04	a"	1371.48	0.00927158
a'	1371.58	0.00197196	a'	1373.3	0.00970268
a'	1397.78	1.26E-04	a"	1399.63	0.0011365
a"	1401.29	2.43E-05	a'	1401.61	0.02764514
a"	1403.64	2.68E-05	a"	1402.69	5.73E-04
a"	1419.23	0.04691565	a'	1420.19	0.31247745
a'	1420.09	0.03915052	a"	1421.68	0.23587357
a'	1442.51	0.02749549	a"	1440.82	0.07692322
a"	1443	0.0189449	a'	1442.04	0.08529624
a'	1487.33	0.37849793	a"	1490.34	1.05001985
a"	1495.02	0.17352761	a'	1510.03	0.19747961
a'	1500.28	0.45815888	a"	1510.19	1.29426463
a"	1509.4	0.00278435	a"	1512.34	0.08187559
a'	1512.78	0.09293995	a'	1514	0.66731616
a"	1513.44	0.2986881	a'	1518.17	1.29772858
a'	1514.46	0.29053388	a"	1520.9	0.00857879
a"	1523.24	0.01846276	a'	1521.21	0.55890525
a'	1536.39	0.26781853	a'	1527.54	50.4338962
a"	1584.2	4.32E-04	a"	1580.16	0.04499892
a'	1584.93	0.0116809	a'	1580.76	0.05206123
a'	3038.89	5.96E-04	a"	3050.2	6.04E-04
a"	3044.84	0.00337267	a'	3050.34	0.00582458
a'	3051.12	0.02716258	a"	3057.1	0.00313416
a"	3052.04	0.01961741	a'	3057.53	0.00341804
a"	3054.84	0.00476228	a"	3058.34	0.00228107
a'	3061.51	0.03007053	a'	3061.94	0.02154958
a'	3064.14	0.36041422	a"	3062.64	0.00359701
a"	3064.17	0.3464523	a'	3069.63	1.55799415
a"	3066.03	0.06093526	a"	3070.35	1.37723714
a"	3074.16	0.25539552	a"	3073.27	2.08173667
a'	3074.45	0.27940788	a'	3073.74	1.84847911
a'	3075.96	0.29917937	a'	3082.96	0.31240528

a"	3078.38	0.0996818	a"	3083.11	0.33635527
a'	3084.78	0.30872365	a"	3086.54	0.14698708
a'	3086.25	0.8643741	a'	3086.92	0.16143826
a"	3087.89	0.06096131	a'	3089.25	4.34708992
a'	3096.8	0.7371704	a'	3094.13	5.39640615

Supplementary Table 4a: Comparison of the experimental and theoretical Raman spectra of the Sm compound **3b**: Description of the vibrational motions attributed to the observed Raman bands (ν denotes a valence stretching vibration, δ an in plane deformation mode, $\delta_{ring\ tilt}$ deformation tilting of a complete ring (with respect to the samarium atom and γ an out of plane deformation mode).

theory			Description of the vibrational mode	experimental
Irrep	ν [cm^{-1}]	Raman Int.		ν [cm^{-1}]
a"	-6.82	0	$\delta_{ring\ tilt}$ (C_8H_8 –Sm– C_9H_9, a“)	70
a'	55.11	4.68397071	$\delta_{ring\ tilt}$ (C_8H_8 –Sm– C_9H_9, a‘)	
a"	59.32	3.41941032		
a'	128.46	100	$\nu_{“sym“}$ (C_8H_8 –Sm– C_9H_9, a‘)	127
a"	130.27	2.16025743		
a"	132.73	50.061167	$\delta_{ring\ tilt}$ (Sm– C_9H_9, a“)	186
a'	133.17	50.2517597	$\delta_{ring\ tilt}$ (Sm– C_9H_9, a‘)	
a'	134.66	0.86943957		
a'	197.72	1.64808609	$\nu_{“antisym“}$ (C_8H_8 –Sm– C_9H_9, a‘)	236
a"	202.69	10.2162119	$\delta_{ring\ tilt}$ (Sm– C_8H_8, a“)	
a'	202.92	8.69031789	$\delta_{ring\ tilt}$ (Sm– C_8H_8, a‘)	
a"	233.55	0.21174406		
a'	236.04	0.19054128		
a"	276.53	5.8533234	δ (C-C-C, C_9H_9, a“)	286
a'	278.11	5.81467298	δ (C-C-C, C_9H_9, a‘)	
a'	354.25	2.78345123	δ (C-C-C, C_8H_8, a‘)	385
a"	357.51	2.71820648	δ (C-C-C, C_8H_8, a“)	
a"	397.44	3.35E-04		
a'	397.6	0.00464328		
a'	490.78	0.1157465		
a"	523.24	0.0058902		
a"	571	0.00319371		
a'	595.49	0.25698278		
a"	599.35	0.00700628		
a'	628.65	0.00115026		
a"	634.32	0.00151834		
a'	652.22	0.17606333	$\gamma_{out\ of\ plane}$ (C-H, C_9H_9, a‘)	not observed
a'	678.44	10.3424552	$\nu_{sym,\ breath}$ (C_9, C_9H_9, a‘)	678
a'	687.52	3.24568551	$\gamma_{out\ of\ plane}$ (C-H, C_8H_8, a‘)	
a"	744.02	0.32031984		
a'	746.77	0.24199156		
a'	748.27	0.44974292		
a'	751.5	9.98572257	$\nu_{sym,\ breath}$ (C_8, C_8H_8, a‘)	748
a"	758.14	0.04851515		
a"	764.53	0.1010815		
a'	766.67	0.0412088		
a"	825.46	0.00704227		
a'	826.5	0.00737195		

a'	854.58	0.31827208		
a"	854.85	0.30795348		
a'	871.03	0.00345319		
a"	874.57	0.00582531		
a'	888.66	0.00724252		
a"	888.77	0.00388517		
a'	889.18	0.00492359		
a'	902.77	0.5761307		
a"	902.96	0.56038686		
a"	914.78	0.00130936		
a"	929.54	1.15E-04		
a'	932.54	0.00274586		
a"	953.85	6.32E-04		
a'	961.43	0.02163546		
a"	963.13	0.0030486		
a"	986.31	0.07959594		
a'	1174.78	0.01393499		
a"	1175.08	0.03256564		
a'	1177.87	0.16788557		
a"	1181.02	0.19999911		
a'	1300.04	0.00136239		
a'	1306.3	0.00509911		
a"	1309.96	0.0027487		
a"	1370.09	8.12E-05		
a'	1370.68	0.00151898		
a"	1371.18	0.00230466		
a"	1398.72	4.46E-05		
a'	1402.45	8.57E-04		
a"	1403.85	2.70E-04		
a'	1423.43	0.31969044		
a"	1424.74	0.2942574		
a"	1441.88	0.09946191		
a'	1443.73	0.10080847		
a'	1449.12	0.40855983		
a'	1504.09	0.87785224	δ (C-C-H, C_8H_8, a‘)	1496
a"	1508.71	0.68307537	δ (C-C-H, C_8H_8, a“)	
a"	1511.72	1.38208961	δ (C-C-H, C_9H_9, a“)	
a'	1512.31	0.0272927		
a'	1513.2	1.35793309	δ (C-C-H, C_9H_9, a‘)	
a"	1517.75	0.10640923		
a'	1526.57	1.01941386	$\nu_{antisym}$ (C_9, C_9H_9, a‘)	1519
a"	1579.14	0.00969044		
a'	1579.36	0.00829025		
a"	1692.9	0.01522605		
a"	3036.14	0.23692889		
a'	3037.31	0.27421148		
a'	3049.46	0.03873287		
a"	3052.58	0.00296243		
a"	3054.42	0.00167391		
a'	3056.4	0.00738604		

a"	3059.84	0.01666108		
a"	3061.18	1.42295623	$\nu_{antisym}$ (C-H, C_9H_9, a“)	3007
a'	3065.78	1.4319717	$\nu_{antisym}$ (C-H, C_9H_9, a‘)	
a"	3068.4	1.67357233	$\nu_{antisym}$ (C-H, C_8H_8, a“)	
a'	3068.47	2.0217453	$\nu_{antisym}$ (C-H, C_8H_8, a‘)	
a"	3076.94	0.65420279		
a'	3077.99	1.01291598		
a"	3081.64	0.23592717		
a'	3082.21	0.28998458		
a'	3084.32	4.03680655	ν_{sym} (C-H, C_9H_9, a‘)	3042
a'	3089.35	5.31999184	ν_{sym} (C-H, C_8H_8, a‘)	

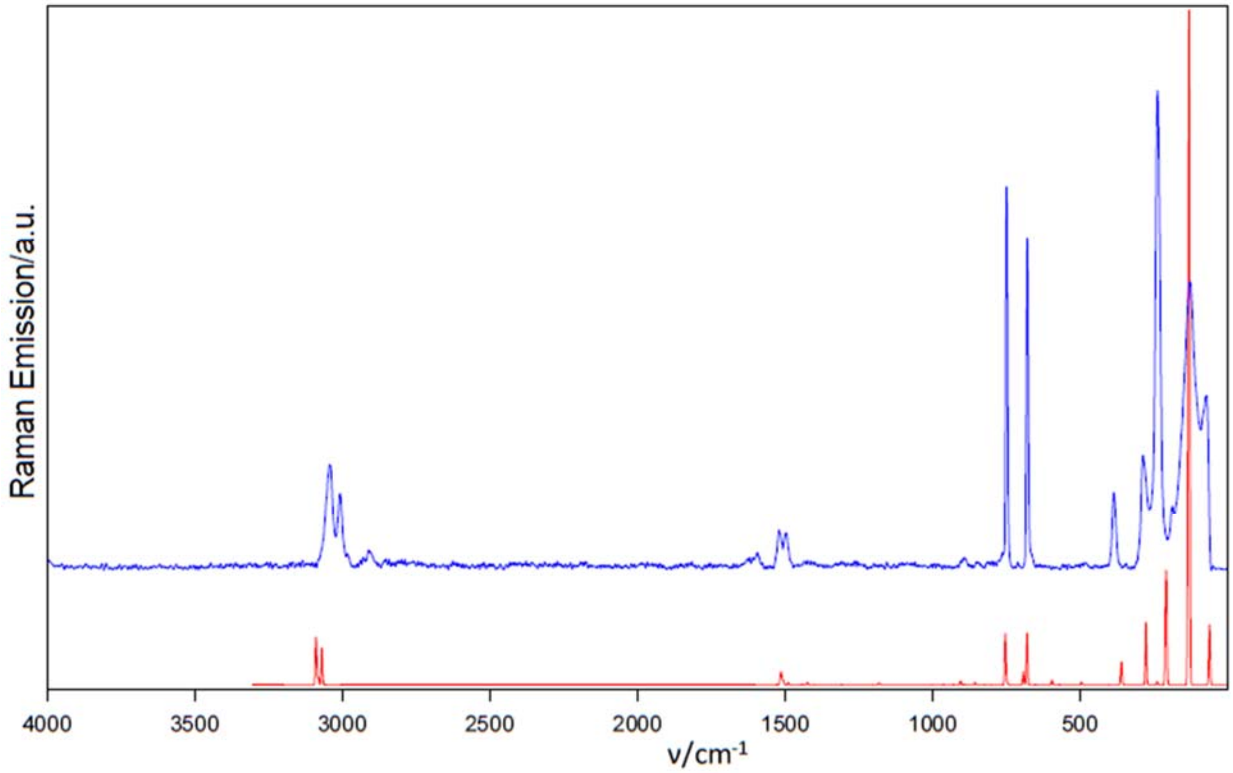

Supplementary Figure 9: Visual comparison of the data presented in Supplementary Table 4a.

Supplementary Table 4b: Comparison of the experimental and theoretical Raman spectra (Metal-Ring vibrations of approximately symmetric and antisymmetric character) of the **3a** to **3d**.

	„sym." metal-ring vibration	„antisym." metal-ring vibration
	$\nu_{exp.}$ ($\nu_{theor.}$) [cm^{-1}]	$\nu_{exp.}$ ($\nu_{theor.}$.) [cm^{-1}]
**Nd (3a)**	137.0 (131.1)	242.1 (221.7)
**Sm (3b)**	126.8 (128.5)	236.0 (197.7)
**Dy (3c)**	100 (127.7)	237.2 (222.4)
**Er (3d)**	165.9 (130.9)	207.4 (190.4)

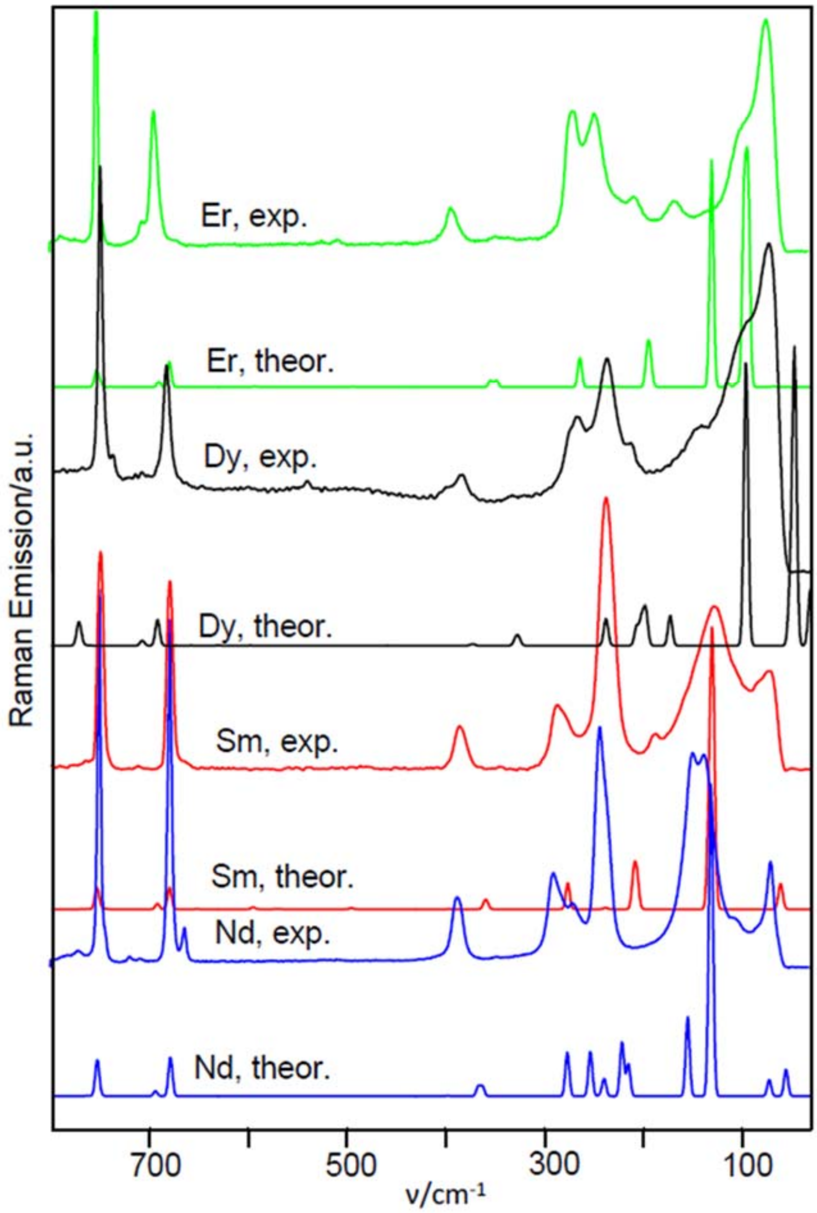

Supplementary Figure 10: Experimental and theoretical Raman spectra of **3a-d**: the depicted frequency range covers the metal ring stretching modes and the ring tilt motions.

Magnetic Studies: Magnetic susceptibility measurements were collected using Quantum Design MPMS®3 and MPMS- XL SQUID magnetometers on polycrystalline material in the temperature range

2 - 300 K under an applied DC magnetic field (H) of 1 kOe. The polycrystalline sample of **3d** was fixed in eicosane and sealed in a quartz tube. Magnetization as a function of applied field was investigated in the field and temperature ranges of 0 – 7 T and 2–10 K, respectively, at a sweep rate of 700 Oe/min. AC data was collected using an oscillating magnetic field of 5 Oe and frequencies between 1 and 1.5 kHz. DC data were corrected for diamagnetic contributions from the eicosane and core diamagnetism employing Pascal's constants.[20]

Supplementary Table 5: Fitting parameters from fitting the out-of-phase component to a modified Debye model under H_{DC} = 0.

Temperature	χ_S	χ_T	τ	α
**2.0**	0.51741	5.53565	1.636354641	0.458492
**3.0**	0.414243	3.62571	1.602482389	0.444562
**4.0**	0.347666	2.69667	1.602482389	0.435746
**5.0**	0.301298	2.16024	1.646622623	0.429774
**6.0**	0.267641	1.82429	1.554237386	0.426041
**7.0**	0.241586	1.6045	1.602482389	0.423886
**8.0**	0.224233	1.3816	1.51257368	0.406515
**9.0**	0.20887	1.17086	1.467035506	0.385757
**10.0**	0.193815	1.01639	1.347606467	0.378869
**11.0**	0.177723	0.944867	1.009670005	0.40762
**12.0**	0.162954	0.961401	0.768563278	0.466466
**13.0**	0.152556	1.03856	0.648521759	0.519395
**14.0**	0.152097	1.05864	0.480082	0.531977
**15.0**	0.159439	0.892846	0.294446	0.483408
**15.5**	0.158515	0.750658	0.225417	0.452741
**16.0**	0.164427	0.743986	0.160595	0.413814
**16.5**	0.16045	0.636955	0.122013	0.37796
**17.0**	0.163794	0.653922	0.0908029	0.344416
**17.5**	0.158721	0.567738	0.0653598	0.301434
**18.0**	0.161258	0.589389	0.0456798	0.269324
**18.5**	0.154668	0.518952	0.0312401	0.232631
**19.0**	0.157222	0.541911	0.0209775	0.205207
**19.5**	0.150329	0.48301	0.0140609	0.182941
**20.0**	0.151942	0.508318	0.00932025	0.167052
**20.5**	0.144764	0.456111	0.00622284	0.15552
**21.0**	0.147738	0.481596	0.00417354	0.144627
**21.5**	0.140687	0.433579	0.00280837	0.136683
**22.0**	0.143598	0.459189	0.00190673	0.131129
**22.5**	0.137559	0.413818	0.0013152	0.121635
**23.0**	0.139919	0.439132	9.06549E-4	0.121511
**23.5**	0.134509	0.396498	6.42604E-4	0.113552
**24.0**	0.138343	0.421022	4.57436E-4	0.105315
**24.5**	0.132781	0.38068	3.32464E-4	0.0961961
**25.0**	0.137296	0.404496	2.43515E-4	0.0907534
**25.5**	0.133104	0.366072	1.84585E-4	0.0793734
**26.0**	0.138446	0.388936	1.39368E-4	0.0695699

Supplementary Table 6: Fitting parameters from fitting the out-of-phase component to a modified Debye model under H_{DC} = 2 kG.

Temperature	χ_S	χ_T	τ	α
**14.0**	3.351494475	3.948501868	2.133637545	0.131482186
**14.5**	3.370929667	3.929070235	1.675198341	0.121594097
**15.0**	3.380534319	3.919465674	1.337244304	0.118989259
**15.5**	3.393040384	3.906959617	1.00085051	0.107913675
**16.0**	3.402809043	3.897190956	0.708622318	0.101102316
**16.5**	3.412191589	3.887808413	0.469876231	0.09397519
**17.0**	3.420361323	3.879638677	0.296311514	0.089384452
**17.5**	3.427686871	3.872313129	0.179611607	0.085885289
**18.0**	3.433940196	3.866059804	0.106913556	0.084444993
**18.5**	3.439787092	3.860212908	0.063362873	0.082482485
**19.0**	3.444734068	3.855265932	0.037852448	0.0829951
**19.5**	3.449214598	3.850785402	0.022894019	0.083369717
**20.0**	3.455520269	3.844479731	0.014255844	0.07638789
**20.5**	3.459748968	3.840251032	0.00891614	0.075845214
**21.0**	3.464532364	3.835467574	0.005734323	0.076861017
**21.5**	3.468430451	3.831569553	0.003725989	0.073439389
**22.0**	3.472570507	3.827429493	0.00246722	0.072735149
**22.5**	3.475842271	3.824157728	0.001677957	0.068780755
**23.0**	3.480339557	3.819660443	0.001139173	0.067450574
**23.5**	3.483408382	3.816591618	7.953994228E-4	0.066098303
**24.0**	3.488547961	3.811452075	5.63417982E-4	0.058430293
**24.5**	3.494375934	3.805624066	4.070393372E-4	0.042881745
**25.0**	3.476006981	3.823993019	2.487730302E-4	0.113817873
**25.5**	3.499569015	3.800430985	2.214917285E-4	0.049353862
**26.0**	3.504433415	3.795566585	1.678784902E-4	0.042051077

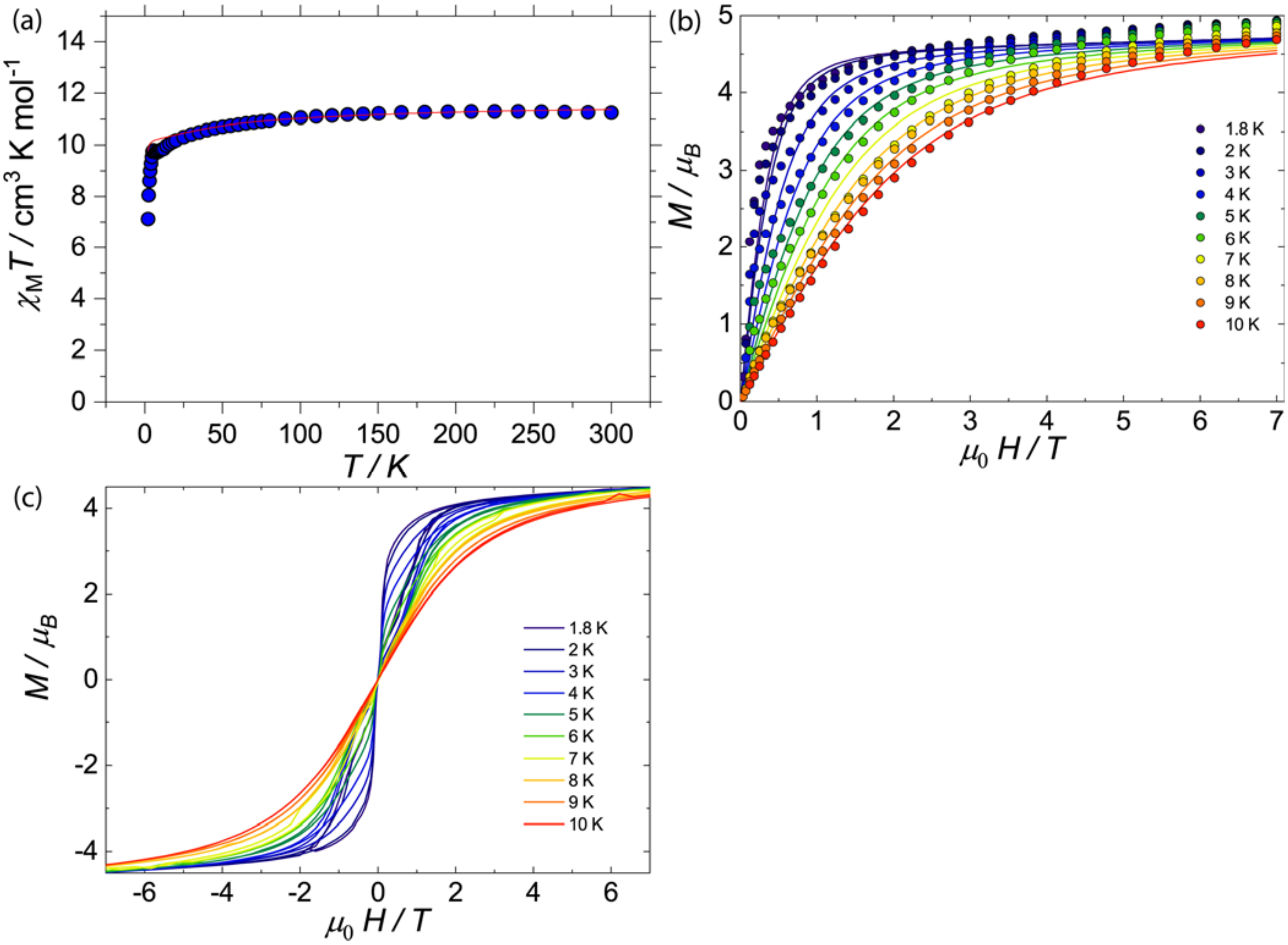

Supplementary Figure 11: (a) Experimental $\chi_M T(T)$ for **3d** under and applied H_{DC} field of 1 kOe and (b) magnetization versus field ($M(H)$) at temperatures from 1.8 K to 10 K in a field range from 0 to 7 T. Solid lines are the calculated $\chi_M T(T)$ and $M(H)$ data employing the crystal field parameters obtained from CASSCF calculations. (c) Hysteresis loops from 1.8 to 10 K in a field range of ±7 T. The small bump at ca. 6 K, can be attributed to intermolecular interaction, not included in the CASSCF calculations.

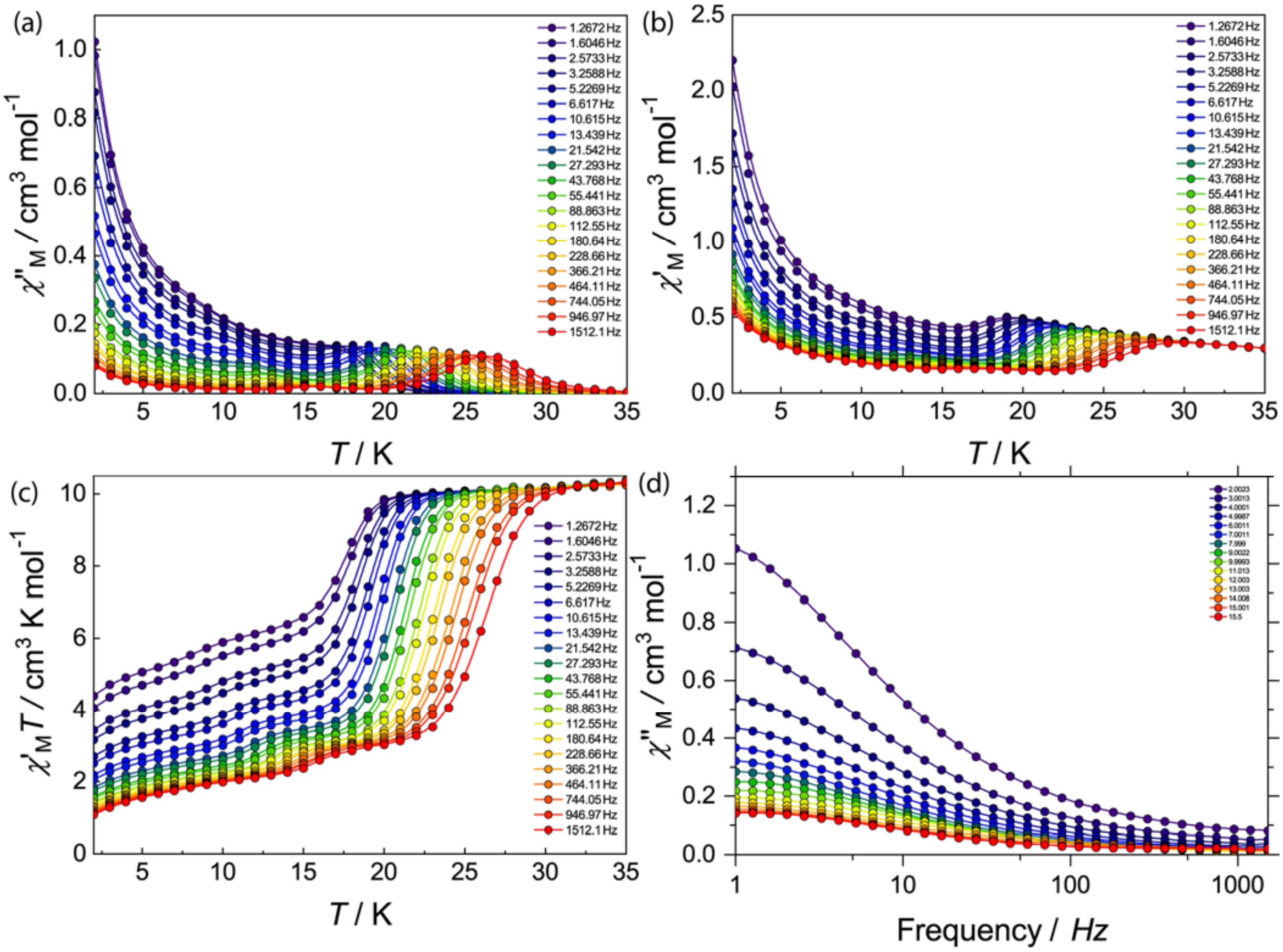

Supplementary Figure 12: Dynamic magnetic data for compound **3d** employing a 5 Oe oscillating field and zero DC field. (a) $\chi_M''(T)$;(b) $\chi_M''(T)$; (c) $\chi_M''T(T)$ and (d) $\chi_M''(\nu)$. Solids lines in (a-c) are a guide to the eye. Note that between 10 K and 15 K a broad peak is observed in the $\chi_M''(T)$, not observable in the $\chi_M''(\nu)$ data. This, could be a result of some degradation of **3d**, resulting in deviation of the ideal fitting, thus resulting in larger α parameters.

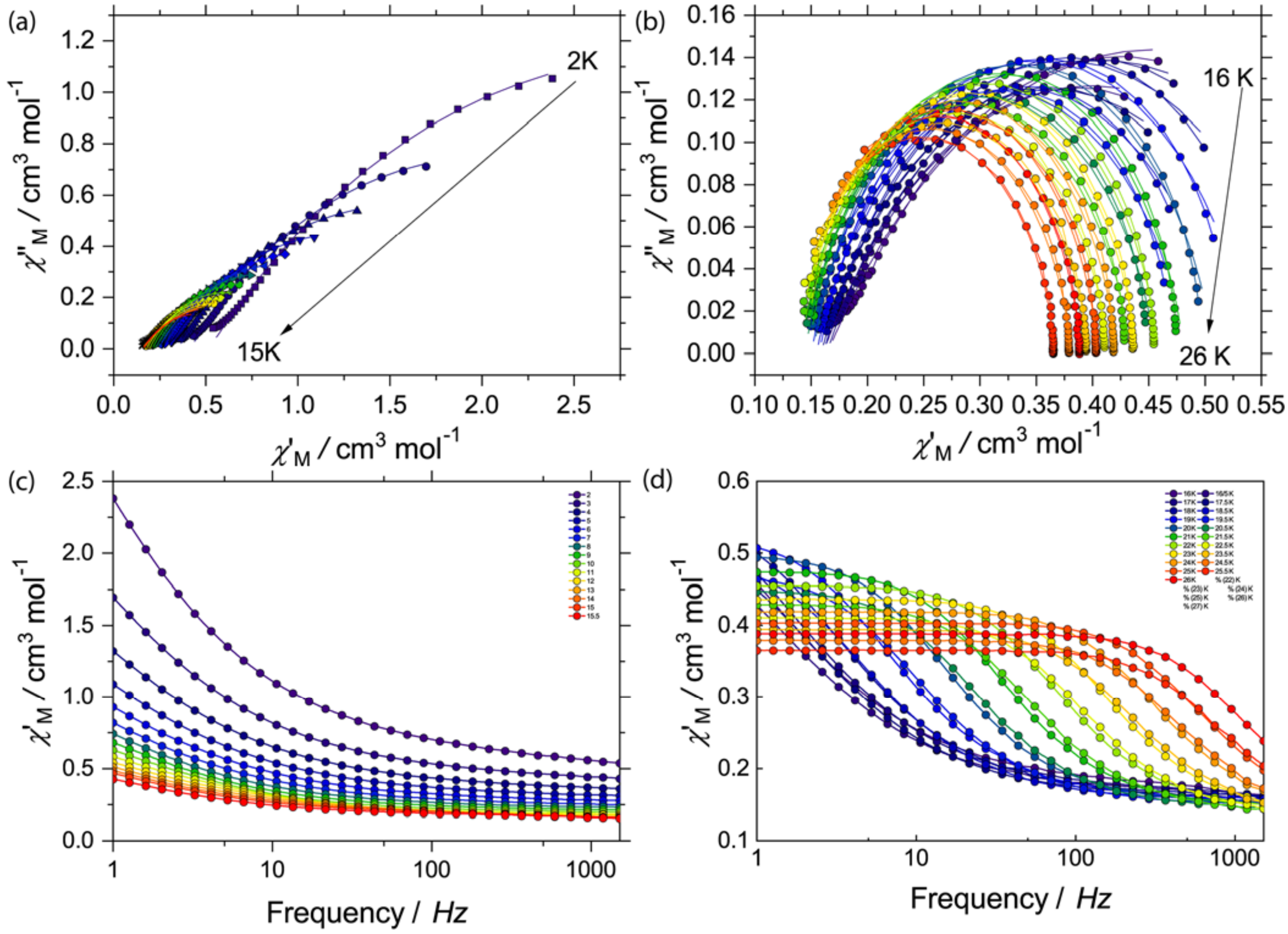

Supplementary Figure 13: Dynamic magnetic data for compound **3d** employing a 3.5 Oe AC field and zero DC field: (a) and (b) are Cole-Cole plots and panels (c) and (d) are $\chi_M'(\nu)$. Panels (a) and (c) correspond to data collected between 2 and 15 K, whilst panels (b) and (d) correspond to the data collected between 16 to 26 K. Solid lines in all panels (a) and (b) are the simulated curves using a generalized Debye model.

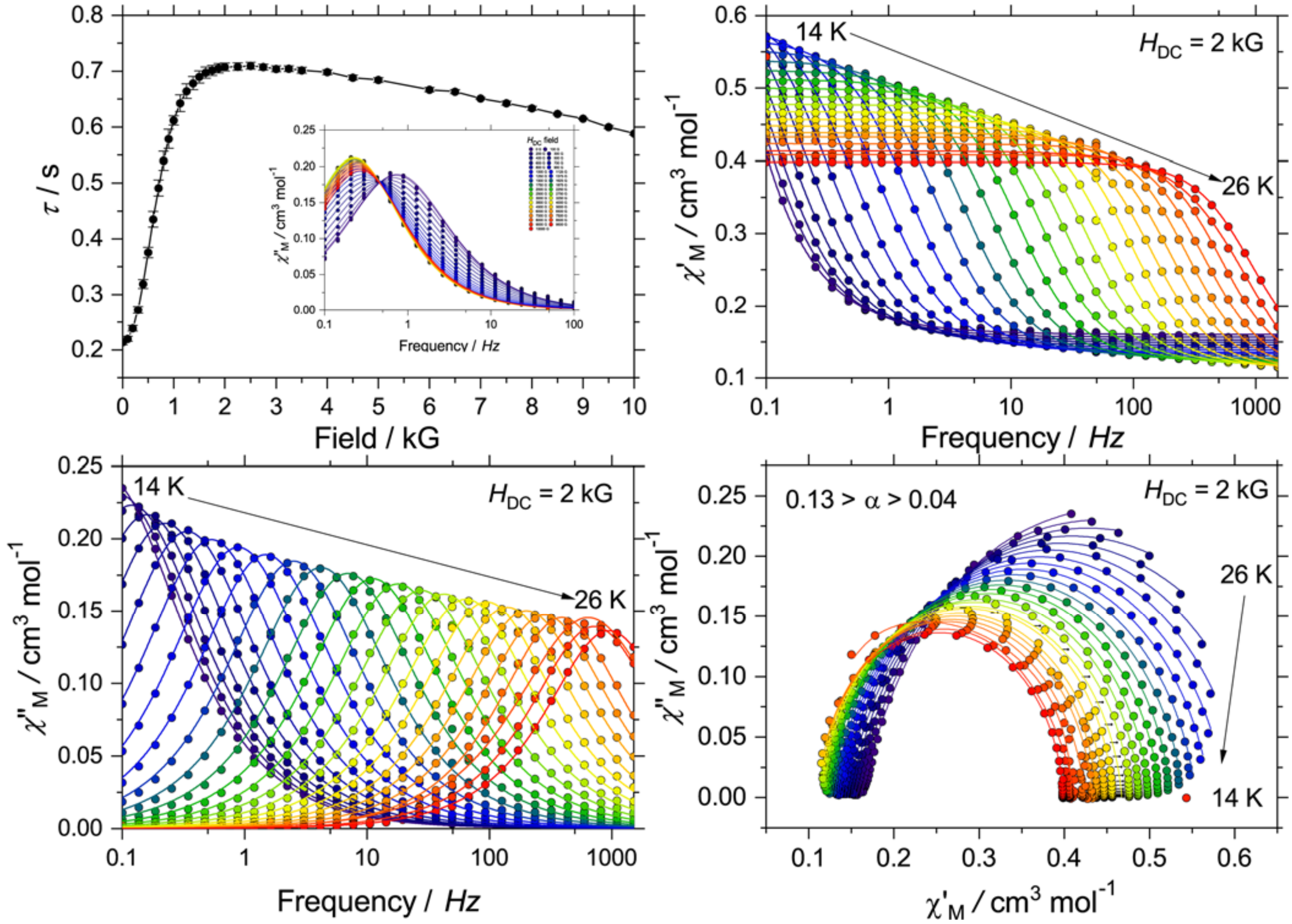

Supplementary Figure 14: Dynamic magnetic data for compound **3d** employing a 3.5 Oe AC field and a H_{DC} = 2 kG: (a) is the τ vs. field study (inset in the $\chi_M''(\nu, H)$). (b) are Cole-Cole plots, whilst (c) and (d) are $\chi_M'(\nu)$ and $\chi_M''(\nu)$, respectively. Solid lines in panels (a) (inset) and (b-d) are the simulated curves using a generalized Debye model.

CASSCF Calculations: *ab initio* calculations were performed with the CASSCF/SO-RASSI/SINGLE_ANISO approach implemented in the Molcas 8.2 program package.[21-26] For this purpose, an optimized crystal structure of **3d** was employed (See geometry optimization details), with the atoms being described using standard basis sets from the ANO-RCC library available in Molcas.[27-29] For the erbium ion a basis set of VTZP quality employed, whilst VDZP quality was used for the C atoms, and VDZ quality for all H atoms, using the second-order DKH transformation.[30] The state-averaged CASSCF orbitals of quartets and doublets were optimized with 35 and 112 states, respectively, with the RASSCF module. The spin-orbit coupling Hamiltonian was then constructed and diagonalized in the basis of 35 and 112, quartets and doublets, respectively, with the RASSI module.[24] The crystal field decomposition of the ground J = 15/2 multiplet of the ^{6}H term was executed with the SINGLE_ANISO module.[25,26]

The energies of low-lying Kramers doublets and the main components of the g tensor of the Er(III) ion are shown in Supplementary Table 7. The main magnetic axis (z) on the Er ions is indicated in Figure 8 by the green arrow, which is perpendicular to the C_8H_8 and C_9H_9 moieties. The ground Kramer doublet state characterize the **3d** is strongly axial, with the following g-values: $g_x = g_y = 0.0000$ and $g_z = 17.9505$, whereas the first excited state lies at 144 cm^{-1} and the second excited state is at 268 cm^{-1}. The ground doublet can be ascribed to an m_J = ±15/2, whilst the first and second excited states are ±13/2 and ±1/2, respectively, with subsequent excited states being highly mixed and bunched over 330 to 490 cm^{-1}. Using the average matrix elements of magnetic moment between the electronic states as a proxy for transition propensity, we predict the most efficient magnetic relaxation pathway to occur *via* the second excited state at 268 cm^{-1} (386 K) (see Figure 8), which is in very close to the U_{eff} obtained from dynamic studies.

Supplementary Table 7: *Ab initio* energy barrier and principal g-tensor for the Kramer doublets of **3d**.

ab initio energy [cm^{-1}]	*ab initio* energy [K]	g_x	g_y	g_z	Angle [º]	Wavefunction
0	0	0.0000	0.0000	17.9505	-	99.99% $\|\pm 15/2\rangle$
143.6	206.6	0.0000	0.0000	15.5401	0.07	99.99% $\|\pm 13/2\rangle$
268.2	385.9	9.6611	9.4615	1.2245	0.04	97.88% $\|\pm 1/2\rangle$
329.8	474.5	0.0367	0.2375	3.6965	1.18	99.96% $\|\pm 3/2\rangle$
349.4	502.7	0.0001	0.0026	13.0490	0.14	99.97% $\|\mp 11/2\rangle$
422.3	607.5	0.1190	0.1580	6.0295	0.12	99.97% $\|\pm 5/2\rangle$
476.1	685.0	0.01381	0.0195	10.7345	0.75	99.97% $\|\mp 9/2\rangle$
490.6	705.9	0.0022	0.0355	8.3874	1.13	99.97% $\|\pm 7/2\rangle$

X-ray Crystallographic Studies: Suitable crystals were covered in mineral oil (Aldrich) and mounted on a glass fiber or MiTeGen holder. The crystals were transferred directly to the cold stream of a STOE IPDS 2 or STOE StadiVari diffractometer.

All structures were solved by using the program SHELXS/T[31,32] and Olex2.[33] The remaining non-hydrogen atoms were located from successive difference Fourier map calculations. The refinements were carried out by using full-matrix least-squares techniques on F^2 by using the program SHELXL.[31,32] In each case, the locations of the largest peaks in the final difference Fourier map calculations, as well as the magnitude of the residual electron densities, were of no chemical significance.

During the refinement process of compounds **3a-d** different disorders of the C_8H_8- and C_9H_9-moieties were encountered. For complexes **3a** and **3b**, both ligand systems are disordered due to rotation of the C_9H_9 and C_8H_8 moiety in the corresponding ligand plane. In the cases of **3c** and **3d**, both ligands are located on the same side of the lanthanide ion and disordered within each other. Moreover, the erbium ion appears to be disordered over two positions as it is not located on the cell´s special position.

Crystallographic data for the structures reported in this paper have been deposited with the Cambridge Crystallographic Data Centre as a supplementary publication no. 1894445-1894450. Copies of the data can be obtained free of charge on application to CCDC, 12 Union Road, Cambridge CB21EZ, UK (fax: (+(44)1223-336-033; email: deposit@ccdc.cam.ac.uk).

Supplementary Table 8: Crystal data and structure refinement for **1**, **2c** and **3a**.

Compound	1	2c	3a
Empirical formula	$C_{17}H_{29}KO_4$	$C_{16}H_{24}DyIO_2$	$C_{17}H_{17}Nd$
Formula weight	336.50	537.75	365.54
Temperature/K	100.0	100.0	150.0
Crystal system	monoclinic	monoclinic	monoclinic
Space group	*C*2/*c*	*P*2_1/*n*	*P*2_1/*n*
a/Å	33.317(3)	8.4875(12)	8.7995(15)
b/Å	7.8873(4)	9.3675(18)	12.029(2)
c/Å	17.0032(13)	22.225(4)	12.7471(19)
β/°	121.100(5)	97.481(12)	90.152(13)
Volume/Å^3	3825.9(5)	1752.0(5)	1349.2(4)
Z	8	4	4
ρ_{calc}g/cm^3	1.168	2.039	1.800
μ/mm^{-1}	0.291	6.026	3.826
F(000)	1456.0	1020.0	716.0
Crystal size/mm^3	0.632 × 0.327 × 0.189	0.378 × 0.29 × 0.191	0.329 × 0.303 × 0.27
Radiation	MoKα (λ = 0.71073)	MoKα (λ = 0.71073)	MoKα (λ = 0.71073)
2Θ range/°	4.792 to 63.644	4.724 to 52	4.656 to 59.3
Index ranges	-48 ≤ h ≤ 42, -11 ≤ k ≤ 11, -23 ≤ l ≤ 24	-10 ≤ h ≤ 10, -11 ≤ k ≤ 11, -26 ≤ l ≤ 27	-10 ≤ h ≤ 12, -16 ≤ k ≤ 16, -17 ≤ l ≤ 17
Refl. collected	22383	11773	15420
Independent refl.	5141 [R_{int} = 0.0289, R_{sigma} = 0.0357]	3442 [R_{int} = 0.0304, R_{sigma} = 0.0272]	3800 [R_{int} = 0.0387, R_{sigma} = 0.0249]
Data/restraints/ parameters	5141/0/203	3442/41/181	3800/298/316
GooF	1.046	1.073	1.123
Final R indexes [I>=2σ (I)]	R_1 = 0.0416, wR_2 = 0.0871	R_1 = 0.0404, wR_2 = 0.0950	R_1 = 0.0178, wR_2 = 0.0496
Final R indexes [all data]	R_1 = 0.0741, wR_2 = 0.1031	R_1 = 0.0506, wR_2 = 0.1008	R_1 = 0.0265, wR_2 = 0.0519
Largest diff. peak/hole/e Å^{-3}	0.297/-0.282	1.679/-1.760	1.05/-0.72

Supplementary Table 9: Crystal data and structure refinement for **3b-d**.

Compound	3b	3c	3d
Empirical formula	$C_{17}H_{17}Sm$	$C_{17}H_{17}Dy$	$C_{17}H_{17}Er$
Formula weight	371.65	383.80	388.56
Temperature/K	100	100.0	100.0
Crystal system	monoclinic	monoclinic	monoclinic
Space group	$P2_1/n$	$P2_1/n$	$P2_1/n$
a/Å	8.751(3)	7.1360(14)	7.0050(8)
b/Å	11.972(5)	8.6865(17)	8.7288(12)
c/Å	12.782(5)	10.874(2)	11.2245(13)
β/°	89.89(3)	100.76(3)	102.421(9)
Volume/Å^3	1339.1(8)	662.2(2)	670.26(14)
Z	4	2	2
ρ_{calc}g/cm^3	1.843	1.925	1.925
μ/mm^{-1}	4.363	5.620	6.238
F(000)	724.0	370.0	374.0
Crystal size/mm^3	0.23 × 0.055 × 0.041	0.072 × 0.063 × 0.058	0.371 × 0.232 × 0.152
Radiation	MoKα (λ = 0.71073)	MoKα (λ = 0.71073)	MoKα (λ = 0.71073)
2Θ range/°	4.662 to 63.068	6.044 to 62.742	5.966 to 58.39
Index ranges	-12 ≤ h ≤ 12, -12 ≤ k ≤ 17, -18 ≤ l ≤ 18	-10 ≤ h ≤ 8, -10 ≤ k ≤ 12, -15 ≤ l ≤ 15	-7 ≤ h ≤ 9, -11 ≤ k ≤ 11, -15 ≤ l ≤ 15
Refl. collected	8152	3606	5760
Independent refl.	3765 [R_{int} = 0.0291, R_{sigma} = 0.0315]	1815 [R_{int} = 0.0418, R_{sigma} = 0.0507]	1806 [R_{int} = 0.0188, R_{sigma} = 0.0212]
Data/restraints/ parameters	3765/258/316	1815/373/160	1806/149/163
GooF	1.065	1.095	1.041
Final R indexes [I>=2σ (I)]	R_1 = 0.0382, wR_2 = 0.1019	R_1 = 0.0480, wR_2 = 0.1310	R_1 = 0.0401, wR_2 = 0.1011
Final R indexes [all data]	R_1 = 0.0598, wR_2 = 0.1164	R_1 = 0.0782, wR_2 = 0.1607	R_1 = 0.0481, wR_2 = 0.1041
Largest diff. peak/hole/e Å^{-3}	1.79/-1.27	2.05/-1.33	1.26/-0.78

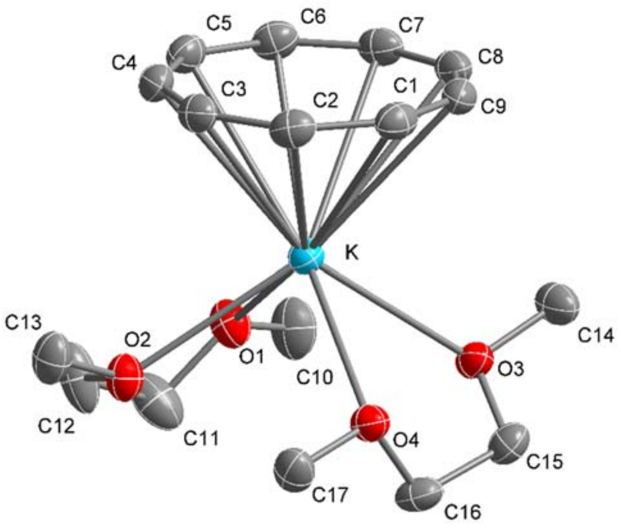

Supplementary Figure 15: Molecular structure of the DME adduct of **1** in the solid state. Hydrogen atoms are omitted for clarity. Selected bond lengths [Å] and angles [°]: K-O1 2.6627(13), K-O2 2.8528(13), K-O3 2.7501(12), K-O4 2.6979(12), K-Centroid 2.3614(5), K-C(C_9H_9) 3.085(2)-3.154(2), C(C_9H_9)-C(C_9H_9) 1.389(3)-1.394(3); C(C_9H_9)-C(C_9H_9)-C(C_9H_9) 139.4(2)-140.4(2).

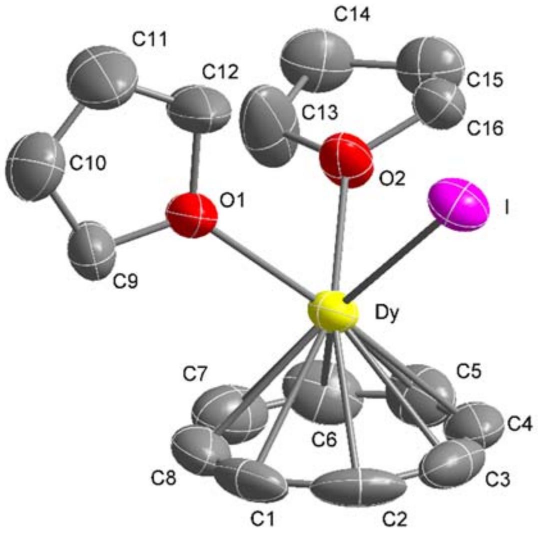

Supplementary Figure 16: Molecular structure of **2c** in the solid state. Hydrogen atoms are omitted for clarity. Selected bond lengths [Å] and angles [°]: Dy-I 3.0535(9), Dy-O1 2.391(6), Dy-O2 2.371(5), Dy-Centroid 1.7945(4), Dy-C(C_8H_8) 2.545(10)-2.575(12), C(C_8H_8)-C(C_8H_8) 1.32(2)-1.46(2); C(C_8H_8)-C(C_8H_8)-C(C_8H_8), 132.7(9)-137.3(11).

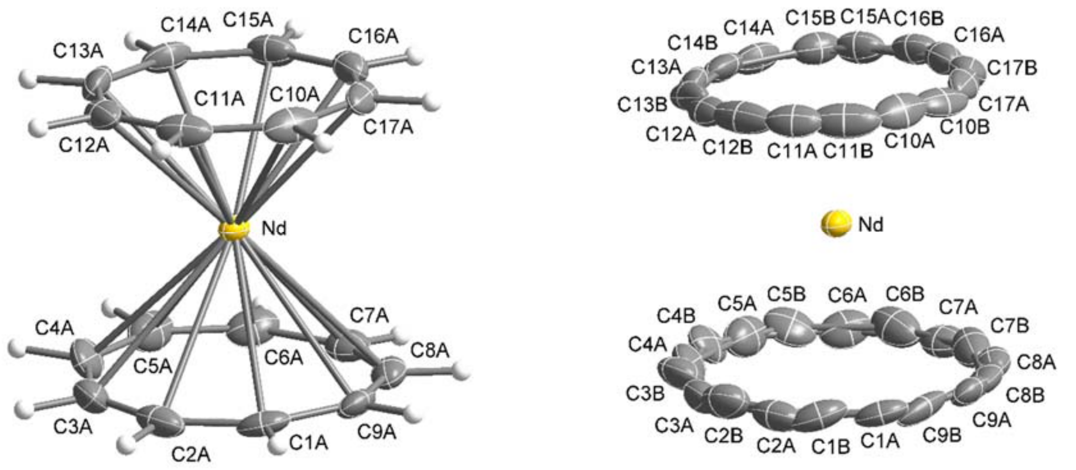

Supplementary Figure 17: Left: Molecular structure of **3a** in the solid state. Only one part of the disordered structure is depicted. Right: Disordered asymmetric unit of **3a**. Hydrogen atoms are omitted for clarity. Selected bond lengths [Å] and angles [°]: Part A: Nd-Centroid(C_9H_9) 2.0441(3), Nd-Centroid(C_8H_8) 1.8925(3), Nd-C(C_9H_9) 2.845(6)-2.915(7), Nd-C(C_8H_8) 2.613(8)-2.653(8), C(C_9H_9)-C(C_9H_9) 1.343(9)-1.423(9), C(C_8H_8)-C(C_8H_8) 1.358(8)1.470(12), C(C_9H_9)-C(C_9H_9)-C(C_9H_9) 138.7(7)-140.7(8), C(C_8H_8)-C(C_8H_8)-C(C_8H_8) 133.5(7)-137.0(7), Centroid(C_9H_9)-Nd-Centroid(C_8H_8) 176.469(13). Part B: Nd-Centroid(C_9H_9) 2.0072(3), Nd-Centroid(C_8H_8) 1.8903(3), Nd-C(C_9H_9) 2.822(9)-2.864(12), Nd-C(C_8H_8) 2.60(2)-2.647(11), C(C_9H_9)-C(C_9H_9) 1.328(12)-1.47(2), C(C_8H_8)-C(C_8H_8) 1.333(8)-1.47(2); C(C_9H_9)-C(C_9H_9)-C(C_9H_9) 137.8(9)-142.5(13), C(C_8H_8)-C(C_8H_8)-C(C_8H_8) 132.1(9)-137.0(10), Centroid(C_9H_9)-Nd-Centroid(C_8H_8) 176.868(13).

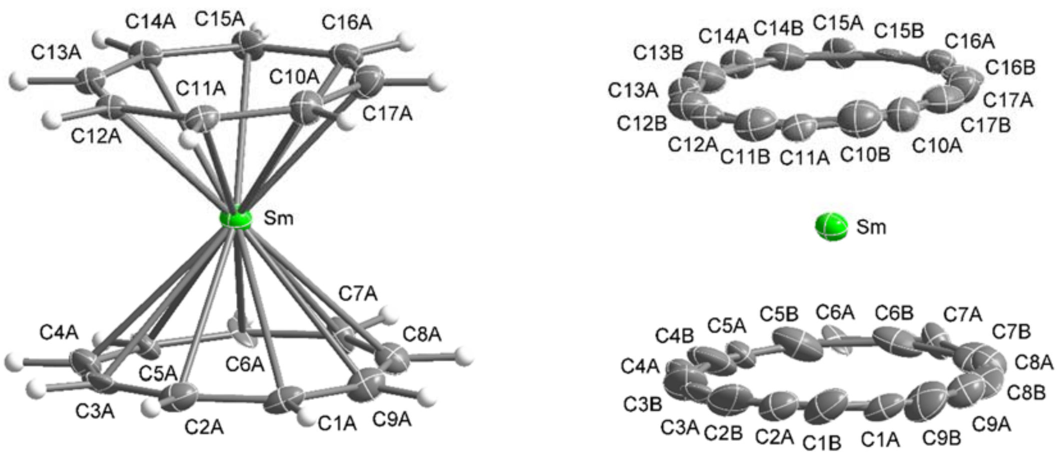

Supplementary Figure 18: Left: Molecular structure of **3b** in the solid state. Only one part of the disordered structure is depicted. Right: Disordered asymmetric unit of **3b**. Hydrogen atoms are omitted for clarity. Selected bond. lengths [Å] and angles [°]: Part A: Sm-Centroid(C_9H_9) 1.9908(6), Sm-Centroid(C_8H_8) 1.8687(5), Sm-C(C_9H_9) 2.814(11)-2.886(12), Sm-C(C_8H_8) 2.623(11)-2.648(11), C(C_9H_9)-C(C_9H_9) 1.34(2)-1.43(2), C(C_8H_8)-C(C_8H_8) 1.372(14)-1.54(2), C(C_9H_9)-C(C_9H_9)-C(C_9H_9) 137.0(11)-142.8(12), C(C_8H_8)-C(C_8H_8)-C(C_8H_8) 133.8(10)-137.7(10), Centroid(C_9H_9)-Sm-Centroid(C_8H_8) 176.30(2). Part B: Sm-Centroid(C_9H_9) 1.9962(6), Sm-Centroid(C_8H_8) 1.8660(5), Sm-C(C_9H_9) 2.79(2)-2.866(14), Sm-C(C_8H_8) 2.579(12)-2.650(13), C(C_9H_9)-C(C_9H_9) 1.34 (2)-1.43(2), C(C_8H_8)-C(C_8H_8) 1.36(2)-1.50(2); C(C_9H_9)-C(C_9H_9)-C(C_9H_9) 138.0(14)-142(2), C(C_8H_8)-C(C_8H_8)-C(C_8H_8) 131.3(11)-138.5(13), Centroid(C_9H_9)-Sm-Centroid(C_8H_8) 177.00(2).

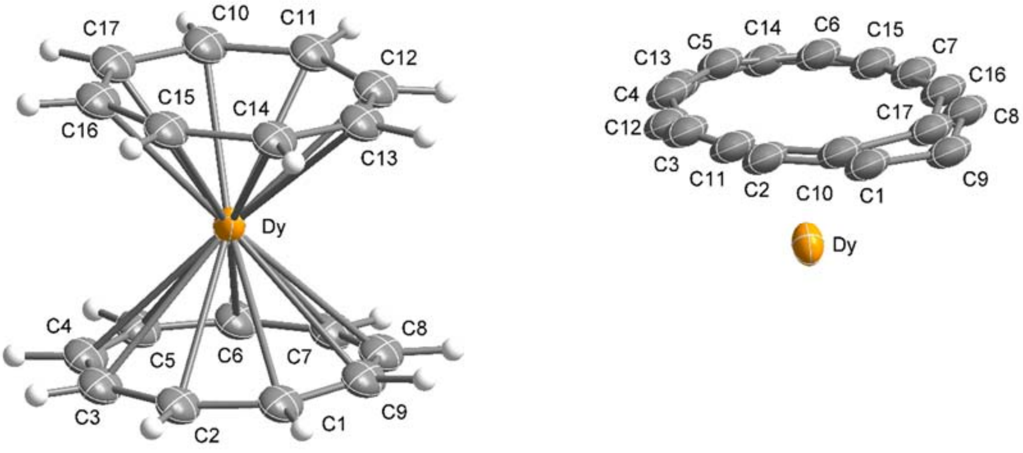

Supplementary Figure 19: Left: Molecular structure of **3c** in the solid state. Only one part of the disordered structure is shown. Right: Disordered asymmetric unit of **3c**. Hydrogen atoms are omitted for clarity. Selected bond lengths [Å] and angles [°]: Dy-Centroid(C_9H_9) 1.8752(3), Dy-Centroid(C_8H_8) 1.8869(4), Dy-C(C_9H_9) 2.707(9)-2.731(9), Dy-C(C_8H_8) 2.61(2)-2.74(2), C(C_9H_9)-C(C_9H_9) 1.345(6)-1.349(6), C(C_8H_8)-C(C_8H_8) 1.41(2)-1.51(2); C(C_9H_9)-C(C_9H_9)-C(C_9H_9) 135.4(12)-141.9(11), C(C_8H_8)-C(C_8H_8)-C(C_8H_8) 133(2)-138(2), Centroid(C_9H_9)-Dy-Centroid(C_8H_8) 172.968(5).

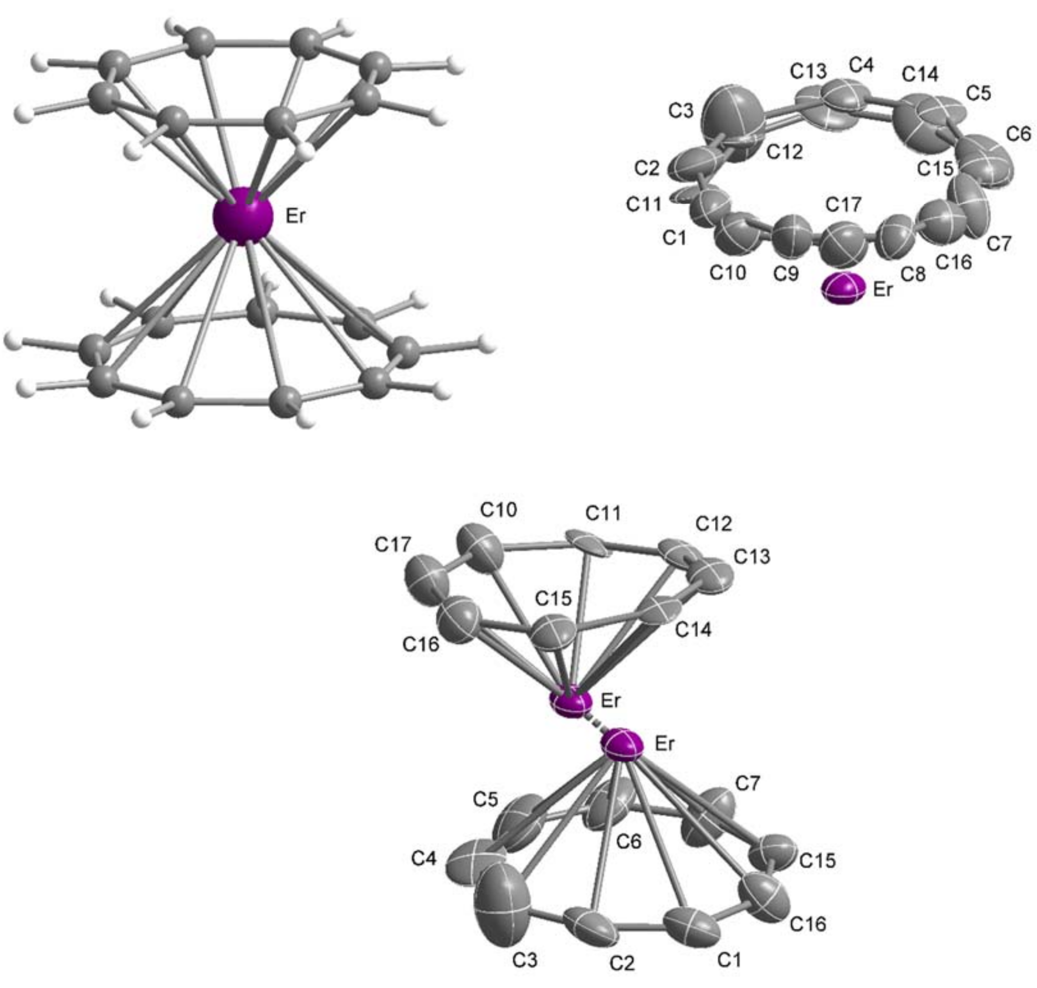

Supplementary Figure 20: Top left: Geometry optimized structure of **3d** (carbon atoms are grey). Top right: Disordered asymmetric unit of **3d** in the solid state. Hydrogen atoms are omitted for clarity. Bottom: Grown asymmetric unit of **3d**. Hydrogen atoms are omitted for clarity. Selected bond lengths [Å] and angles [°]: Er-Centroid(C_9H_9) 1.7248(4), Er-Centroid(C_8H_8) 1.6725(4), Er-C(C_9H_9) 2.468(12)-2.733(10), Er-C(C_8H_8) 2.406(11)-2.670(10), C(C_9H_9)-C(C_9H_9) 1.324(8)-1.362(7), C(C_8H_8)-C(C_8H_8) 1.365(13)-1.52(2); C(C_9H_9)-C(C_9H_9)-C(C_9H_9) 134(2)-149(2), C(C_8H_8)-C(C_8H_8)-C(C_8H_8) 130.5(11)-137.2(11), Centroid(C_9H_9)-Er-Centroid(C_8H_8) 177.516(2).

Supplementary Table 10: Detailed Er-C bond lengths of compound **3d** sorted from short to long.

Er-C(C9H9) [Å]	Er-C(C8H8) [Å]
**2.468(12)**	2.406(11)
**2.521(10)**	2.442(10)
**2.547(13)**	2.448(12)
**2.548(9)**	2.480(10)
**2.621(12)**	2.499(11)
**2.65(2)**	2.560(10)
**2.677(8)**	2.650(12)
**2.726(9)**	2.670(10)
**2.733(10)**	-

Supplementary References

1 Katz, T. J. & Garratt, P. J. Reactions of the Cyclooctatetraenyl Dianion with gem-Dihalides. The Preparation of Derivatives of Bicyclo [6.1.0]nonatriene. Synthesis of the Cyclononatetraenyl Anion. *J. Am. Chem. Soc.* **86**, 5194-5202,(1964).

2 Mashima, K. *et al.* A new convenient preparation of monocyclooctatetraenyl-lanthanide complexes from metallic lanthanides and oxidants. *J. Organomet. Chem.* **473**, 85–91,(1994).

3 Meermann, C., Ohno, K., Törnroos, K. W., Mashima, K. & Anwander, R. Rare-Earth Metal Bis(dimethylsilyl)amide Complexes Supported by Cyclooctatetraenyl Ligands. *Eur. J. Inorg. Chem.* **2009**, 76-85,(2009).

4 Hilgar, J. D., Bernbeck, M. G., Flores, B. S. & Rinehart, J. D. Metal–ligand pair anisotropy in a series of mononuclear Er–COT complexes. *Chem. Sci.* **9**, 7204-7209,(2018).

5 Ahlrichs, R., Bär, M., Häser, M., Horn, H. & Kölmel, C. Electronic structure calculations on workstation computers: The program system turbomole. *Chem. Phys. Lett.* **162**, 165-169,(1989).

6 Treutler, O. & Ahlrichs, R. Efficient molecular numerical integration schemes. *J. Chem. Phys.* **102**, 346-354,(1995).

7 Becke, A. D. Density-functional exchange-energy approximation with correct asymptotic behavior. *Phys. Rev. A* **38**, 3098-3100,(1988).

8 Perdew, J. P. Density-functional approximation for the correlation energy of the inhomogeneous electron gas. *Phys. Rev. B* **33**, 8822-8824,(1986).

9 Perdew, J. P. Erratum: Density-functional approximation for the correlation energy of the inhomogeneous electron gas. *Phys. Rev. B* **34**, 7406-7406,(1986).

10 Sierka, M., Hogekamp, A. & Ahlrichs, R. Fast evaluation of the Coulomb potential for electron densities using multipole accelerated resolution of identity approximation. *J. Chem. Phys.* **118**, 9136-9148,(2003).

11 Weigend, F. & Ahlrichs, R. Balanced basis sets of split valence, triple zeta valence and quadruple zeta valence quality for H to Rn: Design and assessment of accuracy. *PCCP* **7**, 3297-3305,(2005).

12 Weigend, F. Accurate Coulomb-fitting basis sets for H to Rn. *PCCP* **8**, 1057-1065,(2006).

13 Deglmann, P., Furche, F. & Ahlrichs, R. An efficient implementation of second analytical derivatives for density functional methods. *Chem. Phys. Lett.* **362**, 511-518,(2002).

14 Wilson, E. B. D., J. C.; Cross, P. C. *Molecular vibrations : the theory of infrared and Raman vibrational spectra*. (New York: Dover, 1955).

15 Fateley, W. G. & Lippincott, E. R. Vibrational Spectrum and Structure of the Tropylium Ion. *J. Chem. Phys.* **26**, 1471-1481,(1957).

16 Nelson, R. D., Fateley, W. G. & Lippincott, E. R. Normal Coordinate Analysis and Aromatic Character of Five- and Seven-membered Aromatic (CH)n Rings. *J. Am. Chem. Soc.* **78**, 4870-4872,(1956).

17 Goubeau, J. H. Siebert: Anwendungen der Schwingungsspektroskopie in der anorganischen Chemie. Band VÜ der Reihe „Anorganische und allgemeine Chemie in Einzeldarstellungen"Springer-Verlag, Berlin-Heidelberg-New York 1966. . *Berichte der Bunsengesellschaft für physikalische Chemie* **70**, 934-934,(1966).

18 Aleksanyan, V. T. *et al.* Raman spectra of cyclopentadienyl complexes (η^5-$C_5H_5)_2$M (M = Mn, Cr, V, Ru, Os). *J. Organomet. Chem.* **124**, 293-298,(1977).

19 Ito, M. & Shigeoka, T. Raman spectra of benzene and benzene-d_6 crystals. *Spectrochim. Acta* **22**, 1029-1044,(1966).

20 Bain, G. A. & Berry, J. F. Diamagnetic Corrections and Pascal's Constants. *J. Chem. Educ.* **85**, 532,(2008).

21 Aquilante, F. *et al.* Molcas 8: New capabilities for multiconfigurational quantum chemical calculations across the periodic table. *J. Comput. Chem.* **37**, 506-541,(2016).

22 Siegbahn, P. E. M., Almlöf, J., Heiberg, A. & Roos, B. O. The complete active space SCF (CASSCF) method in a Newton–Raphson formulation with application to the HNO molecule. *J. Chem. Phys.* **74**, 2384-2396,(1981).

23 Olsen, J., Roos, B. O., Jo/rgensen, P. & Jensen, H. J. r. A. Determinant based configuration interaction algorithms for complete and restricted configuration interaction spaces. *J. Chem. Phys.* **89**, 2185-2192,(1988).

24 Malmqvist, P. Å., Roos, B. O. & Schimmelpfennig, B. The restricted active space (RAS) state interaction approach with spin–orbit coupling. *Chem. Phys. Lett.* **357**, 230-240,(2002).

25 Ungur, L. & Chibotaru, L. F. Ab Initio Crystal Field for Lanthanides. *Chem. Eur. J.* **23**, 3708-3718,(2017).

26 Chibotaru, L. F. & Ungur, L. Ab initio calculation of anisotropic magnetic properties of complexes. I. Unique definition of pseudospin Hamiltonians and their derivation. *J. Chem. Phys.* **137**, 064112,(2012).

27 Roos, B. O. *et al.* New Relativistic Atomic Natural Orbital Basis Sets for Lanthanide Atoms with Applications to the Ce Diatom and LuF3. *J. Phys. Chem. A* **112**, 11431-11435,(2008).

28 Roos, B. O., Lindh, R., Malmqvist, P.-Å., Veryazov, V. & Widmark, P.-O. Main Group Atoms and Dimers Studied with a New Relativistic ANO Basis Set. *J. Chem. Phys. A* **108**, 2851-2858,(2004).

29 Widmark, P.-O., Malmqvist, P.-Å. & Roos, B. O. Density matrix averaged atomic natural orbital (ANO) basis sets for correlated molecular wave functions. *Theor. Chim. Acta* **77**, 291-306,(1990).

30 Peng, D. & Hirao, K. An arbitrary order Douglas–Kroll method with polynomial cost. *J. Chem. Phys.* **130**, 044102,(2009).

31 Sheldrick, G. A short history of SHELX. *Acta Crystallogr. Sect. A* **64**, 112-122,(2008).

32 Sheldrick, G. Crystal structure refinement with SHELXL. *Acta Crystallogr. Sect. C* **71**, 3-8,(2015).

33 Dolomanov, O. V., Bourhis, L. J., Gildea, R. J., Howard, J. A. K. & Puschmann, H. OLEX2: a complete structure solution, refinement and analysis program. *J. Appl. Crystallogr.* **42**, 339-341,(2009).
